# High-resolution age-specific mapping of the two-week illness prevalence rate based on the National Health Services Survey and geostatistical analysis: a case study in Guangdong province, China

**DOI:** 10.1186/s12942-021-00273-1

**Published:** 2021-05-03

**Authors:** Chuchun Wen, Xiaoliang Huang, Lifen Feng, Long Chen, Wei Hu, Yingsi Lai, Yuantao Hao

**Affiliations:** 1 School of Public Health, Sun Yat-sen University, Guangzhou, 510080 China; 2 Department of Statistics, Government Affairs Service Center of Health Commission of Guangdong Province, Guangzhou, 510060 China

**Keywords:** Two-week illness, Bayesian geostatistical model, High resolution mapping

## Abstract

**Background:**

The two-week illness prevalence rate is an important and comparable indicator of health service needs. High-spatial-resolution, age-specific risk mapping of this indicator can provide valuable information for health resource allocation. The age-prevalence relationships may be different among areas of the study region, but previous geostatistical models usually ignored the spatial-age interaction.

**Methods:**

We took Guangdong province, the province with the largest population and economy in China, as a study case. We collected two-week illness data and other potential influencing predictors from the fifth National Health Services Survey in 2013 and other open-access databases. Bayesian geostatistical binary regression models were developed with spatial-age structured random effect, based on which, high-resolution, age-specific two-week illness prevalence rates, as well as number of people reporting two-week illness, were estimated. The equality of health resource distribution was further evaluated based on the two-week illness mapping results and the health supply data.

**Results:**

The map across all age groups revealed that the highest risk was concentrated in the central (i.e., Pearl River Delta) and northern regions of the province. These areas had a two-week illness prevalence > 25.0%, compared with 10.0–20.0% in other areas. Age-specific maps revealed significant differences in prevalence between age groups, and the age-prevalence relationships also differed across locations. In most areas, the prevalence rates decrease from age 0 to age 20, and then increase gradually. Overall, the estimated age- and population-adjusted prevalence was 16.5% [95% Bayesian credible interval (BCI): 14.5–18.6%], and the estimated total number of people reporting illness within the two-week period was 17.5 million (95% BCI: 15.5–19.8 million) in Guangdong Province. The Lorenz curve and the Gini coefficient (resulted in 0.3526) showed a moderate level of inequality in health resource distribution.

**Conclusions:**

We developed a Bayesian geostatistical modeling framework with spatial-age structured effect to produce age-specific, high-resolution maps of the two-week illness prevalence rate and the numbers of people reporting two-week illness in Guangdong province. The methodology developed in this study can be generalized to other global regions with available relevant survey data. The mapping results will support plans for health resource allocation.

**Supplementary Information:**

The online version contains supplementary material available at 10.1186/s12942-021-00273-1.

## Background

Decision making in health services should be based on accurate information of supply and need, for optimization of health resource allocation. The accurate supply information (e.g., number of medical staffs, number of hospital beds) are usually recorded by health facilities and easy to obtain, while common methods to estimate the health need have certain limitations. Many researchers used population number as an indicator for health need [1, 2], which is simple, but too crude considering the heterogeneity of illness risk in different places. Others used the demand indicators such as the number of visits to hospital [3, 4], which is an important indicator for hospital planning [5, 6]. However, the demand indicators may not fully represent the need, as gaps usually exist due to barriers like lack of money of patients to hospital, low geographical accessibility and cultural differences between patients and health providers [7]. And there may be “supplier-introduced demand” [8]. In this regard, the prevalence of illness within a two-week period, known as the two-week illness prevalence rate, directly reflects the risk of illness, breaks through the above limitations, and becomes an important and comparable indicator for health service needs [9–11]. This indicator is usually estimated by the self-reported illness status in health surveys, and the time frame “two-week” is considered reasonable for accurate recall of information about the illness and treatment [12, 13]. Till now, two-week illness has been applied in various health surveys worldwide [10, 14–18].

As spatial difference of two-week illness prevalence rate may exist within administrative divisions, obtaining it at high-spatial resolution is helpful for local spatial-targeting allocation of health resources. With a limited number of survey locations, this indicator has often been reported at a low spatial resolution [e.g., cities or provinces), as it is difficult to estimate for smaller spatial units using common statistical approaches [19]. Today, a Bayesian geostatistical modeling framework can be applied to overcome this challenge. This framework can be used to map the disease risk in areas without observed data, via extrapolation based on available disease data from adjacent areas and information about potential influencing predictors [20]. In this way, explicit spatial information of the disease is obtained despite the limited availability of survey locations. Accordingly, the Bayesian geostatistical modeling framework plays an important role in both the implementation of interventions and the study of supply-and-need relationships.

Disease occurrence can be affected by a wide range of variables, including demographic factors (e.g., age [21] and gender [21]), socioeconomic factors (e.g., ethnicity [22], marital status [23], education level [24], employment status [24], occupation [25], income [24] and urbanization level [26]) and climatic and environmental factors (e.g., temperature [27], precipitation [28], landcover [29], elevation [30] and air pollution [27]). Particularly, age is a main factor related to disease occurrence; different age groups are susceptible to different diseases and have different levels of disease risk. Furthermore, a previous report showed that different age groups report different levels of satisfaction with health services, and suggested that services for children and the elderly should be strengthened to promote equity in health service utilization [31]. This suggests that an age-specific analysis of the distribution of illness prevalence is necessary to inform management measures specific to different age groups.

Most previous statistical models addressed the age effect on disease risk either using a linear or categorical form of age as a fixed effect, or applying spline methods [32–34]. However, such models are not able to deal with situations where the age-prevalence relationship differs across the study region. Even though the two-week illness prevalence usually shows a pattern of a higher risk in young children and elderly people and a lower risk in other age groups [35], different areas may not have the exact same age-prevalence relationship. Geostatistical models can, however, be developed to address spatial-age interaction issue, allowing to estimate the different age-prevalence relationships among different areas. Parsaeian et al. used area-specific exponential functions to account for the variation of disease burden with age [36], which may call for high computational burden when interaction with space at high resolution. Based on the Bayesian geostatistical modeling framework, setting and projecting mesh knots is a considerable method to lower the computational burden for space-factor interaction [34].

This study aimed to develop a geostatistical modeling framework to address spatial-age interaction of the two-week illness prevalence and to provide high-spatial-resolution, age-specific estimates, based on data from health services survey. We chose Guangdong province, China as a representative region for case study. To further show the application potential of the outcomes, we evaluated the equality of health resource distribution based on the two-week illness mapping results and the health supply data.

## Methods

### Study area

**Fig. 1:**
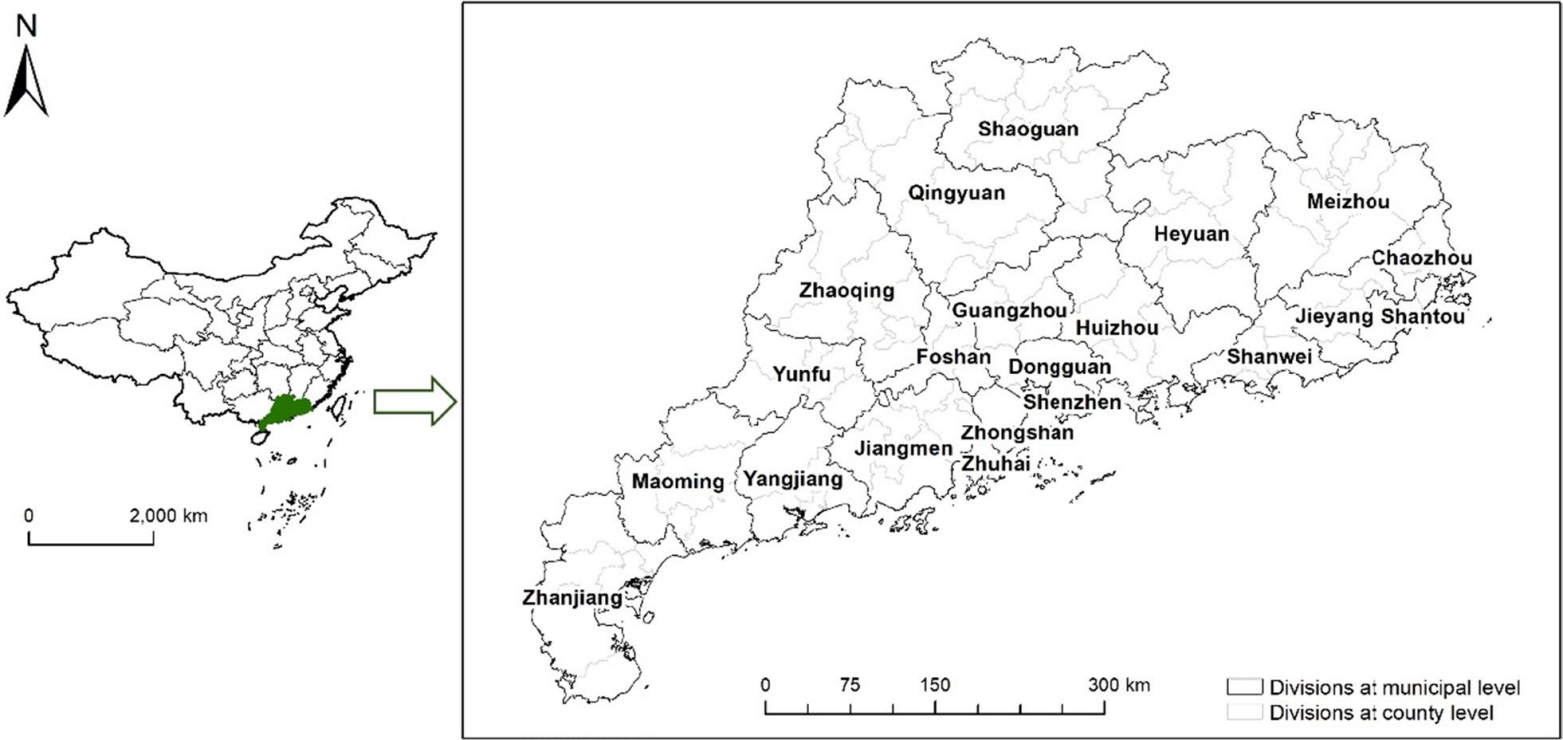
Location of Guangdong province and the names of its municipalities

Guangdong province occupies a region of south China with a longitude ranging from 109° 39ʹ to 117° 19ʹ E and latitude from 20° 13ʹ to 25° 31ʹ N (Fig. 1), having a subtropical to tropical climate. The province is divided into 21 prefecture-level cities and further subdivided into 119 county-level divisions. In 2018, Guangdong had a population of 11.3 million and a gross domestic product (GDP) of 972.7 million yuan, making it the province with both the largest provincial population and economy in China [37]. The five most prevalent diseases in Guangdong province in 2013 were reported as hypertension, acute nasopharyngitis, diabetes, upper respiratory tract infection and gastroenteritis [38], and the raw prevalence are 6.09%, 2.15%, 1.27%, 1.86%, 0.78%, respectively, in the current study dataset, which contributes greatly to the two-week illness. Since 2005, Guangdong has faced the increasing pressure imposed by an aging population [39], bring the age effect to forefront on illness and health services. The province shows large geographic heterogeneity in disease risk, demographic characteristics (e.g., age structure), socioeconomic characteristics (e.g., ethnic composition, income and urbanization level), environmental characteristics (e.g., temperature, landcover and elevation), as well as health service provision [40]. To certain extent, it represents a typical large region to study the health service need.

### Data source

The data used in this study were obtained mainly from the fifth National Health Services Survey of Guangdong province, China. The original individual-level dataset is not publicly available due to the confidentiality required by the fifth National Health Services Survey. This large-scale survey was administered during September and October 2013 and was subdivided into three sub-surveys administered to household residents, medical staff and medical institutions. For the household survey, a multi-stage sampling design was applied to select representative participants randomly. Information was collected on various topics, including demographics; socioeconomic factors; the need, demand and utilization of health services; and the level of satisfaction of health services [38]. Qualified investigators visited the sampled households, inquired about all members of each household and completed the questionnaires [38]. Quality control measures were used throughout the investigation, and the survey was rated as being of good quality, with a Myer’s index of 2.38 and Gini concentration ratio of 0.3048 [38].

The two-week illness status was used as the dependent variable in this study. Respondents were defined as having had an illness within the previous two-week period if they met one of the following three circumstances during the 14 days prior to the interview: (i) visiting a doctor because of illness or injury; (ii) receiving medical treatment because of illness or injury; or (iii) missing work or being bedridden because of illness or injury for at least 1 day [38]. Other demographic and socio-economic factors that might have influenced the two-week illness status (i.e., age, gender, ethnicity, marital status, education level, employment status, occupation, type of household registration and location of household registration) were also obtained from the survey and treated as independent variables in this study.

The coordinates of the survey villages/communities (Fig. 1) were extracted from BaiduMap and transformed to WGS84 using the convBD2WGS function in the madlogos/aseshms package on the R platform [41]. Other environmental and socio-economic factors with potential effects on the outcomes were extracted from different open-access sources (Additional file 1: Table S1, Figure S1). As communities/villages in China usually cover areas larger than 1km^2^ and many risk mapping studies usually took the spatial resolution of 5 × 5 km^2^ [20, 33, 34], we adopted this resolution for this risk mapping study. We aligned the covariate data over a regular grid of 5 × 5 km^2^ spatial resolution and further assigned to the survey villages/communities. For covariates with resolution higher than 5 × 5 km^2^, we linked each pixel of the grid with the aggregated value of the corresponding covariate within the square of 5 × 5 km^2^ centered by the pixel. The arithmetic mean and the mode were used to calculate the aggregated value for continuous and categorical covariates, respectively. For covariates with resolution lower than 5 × 5 km^2^, we assigned each pixel of the 5 × 5 km^2^ the value of the corresponding covariate closest to the pixel. Altogether, we considered 8 individual-level variables (age, gender, ethnicity, education level, marital status, occupation, type of household registration and location of household registration) and 19 location-level variables [living space per capita, proportion of no housing population, proportion of households with unimproved drinking water, proportion of households without sanitary toilets, family population, travel time to cities, GDP per capita, salary per capita, nighttime light, landcover, land surface temperature (LST) in the daytime and at night, normalized difference vegetation index (NDVI), soil moisture, elevation, air temperature, precipitation, fire emissions indicators (total carbon content, TCC) and particulate matter < 2.5 µm in diameter (PM_2.5_)] as potential influencing factors in the subsequent geostatistical analysis. Furthermore, we used the numbers of health workers indicating the supply of health service, for the subsequent analysis of equality, which was obtained from the Guangdong Health Statistics Yearbook (2013) at county level [42] (Additional file 1: Figure S2).

### Statistical analysis

First, we conducted a univariate analysis to identify individual-level variables associated with the two-week illness status, and excluded those that did not differ significantly (significance level $\alpha =0.05$). Continuous variables were standardized, and the ratio variables were log transformed. To avoid collinearity, we dropped one continuous variable in each pair if the corresponding Pearson’s correlation coefficient was > 0.8, and kept the other one which was more meaningful on the outcome variable or with better data quality. To identify the best functional forms (continuous or categorical) of continuous predictors, we converted them to categorical ones according to a preliminary exploratory graphical analysis. And we further built two univariate geostatistical models for each continuous variable, one with the corresponding variable as the only fixed effect variable in a continuous form, and the other with it in a categorical form (treated as dummy variables) [43, 44]. The functional form with the smallest log-scores was included in the multiple geostatistical models, and the best set of covariates was selected using the backward elimination approach [45].

Bayesian geostatistical binary regression models were developed to evaluate the correlations of potential influencing factors (covariates) with the two-week illness status. For the ${i}^{th}$ individual belonging to the ${j}^{th}$ location, we assumed an illness status ${Y}_{ij}$ (${Y}_{ij}=1$ indicating illness and ${Y}_{ij}=0$ indicating no illness within the previous two weeks) according to a Bernoulli distribution, where ${p}_{ij}$ indicates the two-week illness prevalence rate:

1 $${Y}_{ij}\sim Bernoulli\left({p}_{ij}\right)$$

Here, the logit transformation of ${p}_{ij}$ is assigned to a linear combination of effects:

2 $$\text{logit}\left({p}_{ij}\right)={\beta }_{0}+{{\varvec{X}}}_{ij}^{T}{\varvec{\beta}}+{\varphi }_{j(a)}+{\omega }_{j}$$

Here ${{\varvec{X}}}_{ij}$, ${\beta }_{0}$ and ${\varvec{\beta}}$ are denoted as the matrix of covariates, the intercept and the vector of regression coefficients, respectively. These make up the fixed effects (level-1) of the model. A spatial-age random effect ${\varphi}_{j(a)}$ is introduced into the model to describe the age- and location-specific variations, with $a$ the year of age. It is assumed to follow a zero-mean Gaussian process, whose covariance is defined as the Kronecker product of the spatial covariance matrix ${{\varvec{K}}}_{{\varvec{s}}}$ and the age covariance matrix ${{\varvec{K}}}_{{\varvec{a}}}$:

3 $$\boldsymbol{\varphi }\sim GP(0,{{\varvec{K}}}_{{\varvec{s}}}\otimes {{\varvec{K}}}_{{\varvec{a}}})$$

${{\varvec{K}}}_{{\varvec{s}}}$ is defined as the stationary Matérn covariance function, that is ${{\varvec{K}}}_{s}=\frac{{\sigma }^{2}}{{2}^{v-1}\Gamma \left(v\right)}{\left(\kappa {\varvec{D}}\right)}^{v}{K}_{v}(\kappa {\varvec{D}})$, and ${\sigma }^{2}=\frac{1}{4\pi {\kappa }^{2v}{\tau }^{2}}$. Here ${\varvec{D}}$ is the Euclidean distance matrix,${K}_{\nu }$ the modified Bessel function of the second kind with the smoothness parameter $\nu$ fixed at 1, *κ* a scaling parameter for the range $r=\sqrt{8\nu }/\kappa$, and $\tau$ the precision parameter for the spatial variance ${\sigma }^{2}$.${{\varvec{K}}}_{{\varvec{a}}}$ is defined as autoregressive stochastic process with the first order (AR1). In order to reduce computational burden, the Gaussian Markov Random Fields (GMRF) are built on regular age knots, i.e., $\boldsymbol{\varphi}={\left(\boldsymbol{\varphi}_{a=0},\boldsymbol{\varphi}_{a=10},\dots ,\boldsymbol{\varphi}_{a=90},\boldsymbol{\varphi}_{a=\text{max}(age)}\right)}^{\prime}$, and the random fields of other ages are approximated by the projection of $\boldsymbol\varphi$ using the B-spline basis function of degree two. ${\omega }_{j}$ is the unstructured random effect, assumed to follow a zero-mean normal distribution:

4 $${\omega }_{j}\sim Normal(0,{\delta }^{2})$$

which describes the unexplained random variance separately from the variance explained by fixed effect predictors and spatial-age random effect. ${\varphi}_{j(a)}$ and ${\omega }_{j}$ make up the random effects (level-2) of the model.

The following less-informative priors were used for the parameters and hyperparameters: ${\beta }_{0}{,\boldsymbol{ }{\varvec{\beta}}}_{{\varvec{i}}{\varvec{j}}},{{\varvec{\beta}}}_{{\varvec{j}}}\sim Normal (0, 1000)$, $\text{log}\left(\tau \right)\sim Normal (1, 10)$,$\text{log}\left(\kappa \right)\sim Normal (1, 10)$, ${1/\delta }^{2}\sim gamma (1, 0.00005)$ and $\text{log}\left((1+\rho )/(1-\rho )\right)\sim N(\text{0,0.15})$. Sensitivity analyses on priors of hyperparameters was done, by setting different priors for each hyperparameter and comparing the posterior estimates. The model was fitted using the integrated nested Laplace approximations (INLA) with the homonymous package on the R [46].

We calculated estimates over the grid of 5 × 5 km^2^ resolution across Guangdong province, to produce the two-week illness prevalence maps at a high spatial resolution. Age-specific maps were developed first, and then a map of the overall two-week illness prevalence was produced by weighting the age-specific maps according to the population composition across the age groups. We also estimated the number of people who reported illness within the two-week period by multiplying the estimated two-week illness prevalence with the gridded population. The age- and population-adjusted two-week illness prevalence and the total number of people reporting illness within the two-week period were also estimated at the county- and municipality-levels and across the entire province, by combining the prevalence maps with demographic and administrative maps. In addition, we run separate models for the major types of diseases reported (i.e., endocrine and metabolic diseases, circulatory system diseases, respiratory diseases, and digestive system diseases), under the same modeling framework.

We applied fivefold out-of-sample cross-validation to evaluate the model performance. The observed and estimated prevalence (${\widehat{\pi }}_{j}$) in the validation set (with $N$ locations) were compared. Indicators were calculated, namely, the mean error ($ME=\frac{1}{N}\sum_{j=1}\left({\pi }_{j}-{\widehat{\pi }}_{j}\right)$), the mean absolute error ($MAE=\frac{1}{N}\sum_{j=1}\left|{\pi }_{j}-{\widehat{\pi }}_{j}\right|$), the proportion of locations with observed prevalence rates within the 95% Bayesian credible intervals (BCIs), and the area under the receiver operating characteristic (ROC) curve (AUC) [47].

The Lorenz curve and Gini coefficient were used to evaluate the equality of health resource distribution, based on the estimated number of people reporting illness (need) and the number of health workers (supply) at county level:

5 $$G=1-{\sum }_{K=1}^{n-1}\left({F}_{K+1}-{F}_{K}\right)({\phi }_{K+1}+{\phi }_{K})$$

6 $${F}_{K}={\sum }_{k=1}^{K}\frac{{m}_{k}}{M}$$

7 $${\phi }_{K}={\sum }_{k=1}^{K}\frac{{h}_{k}}{H}$$

Here ${m}_{k}$ is denoted be the number of people reporting two-week illness at each county $k$ (the sum of which is$M$) and ${h}_{k}$ the number of health workers at the county$k$. All the counties were ranked according to the supply-need ratio${h}_{k}/{m}_{k}$. Next, the cumulative proportion of the patients up to county $K$ (${F}_{K}$) and the cumulative proportion of health workers (${\phi }_{K}$) is calculated (define${F}_{0}={\phi }_{0}=0$). The Gini coefficient ($G$) was obtained by formula (5), and the Lorenz curve was graphed with (${F}_{K} ,{\phi }_{K}$). A map of the supply-need ratio was also produced to identify counties with relatively less health resources.

All statistical analyses were conducted using R software (version 3.6.1; The R Project for Statistical Computing, Vienna, Austria), and the maps were visualized using ArcGIS (version 10.2; ESRI, Redlands, CA, U.S.A.).

## Results

**Fig. 2:**
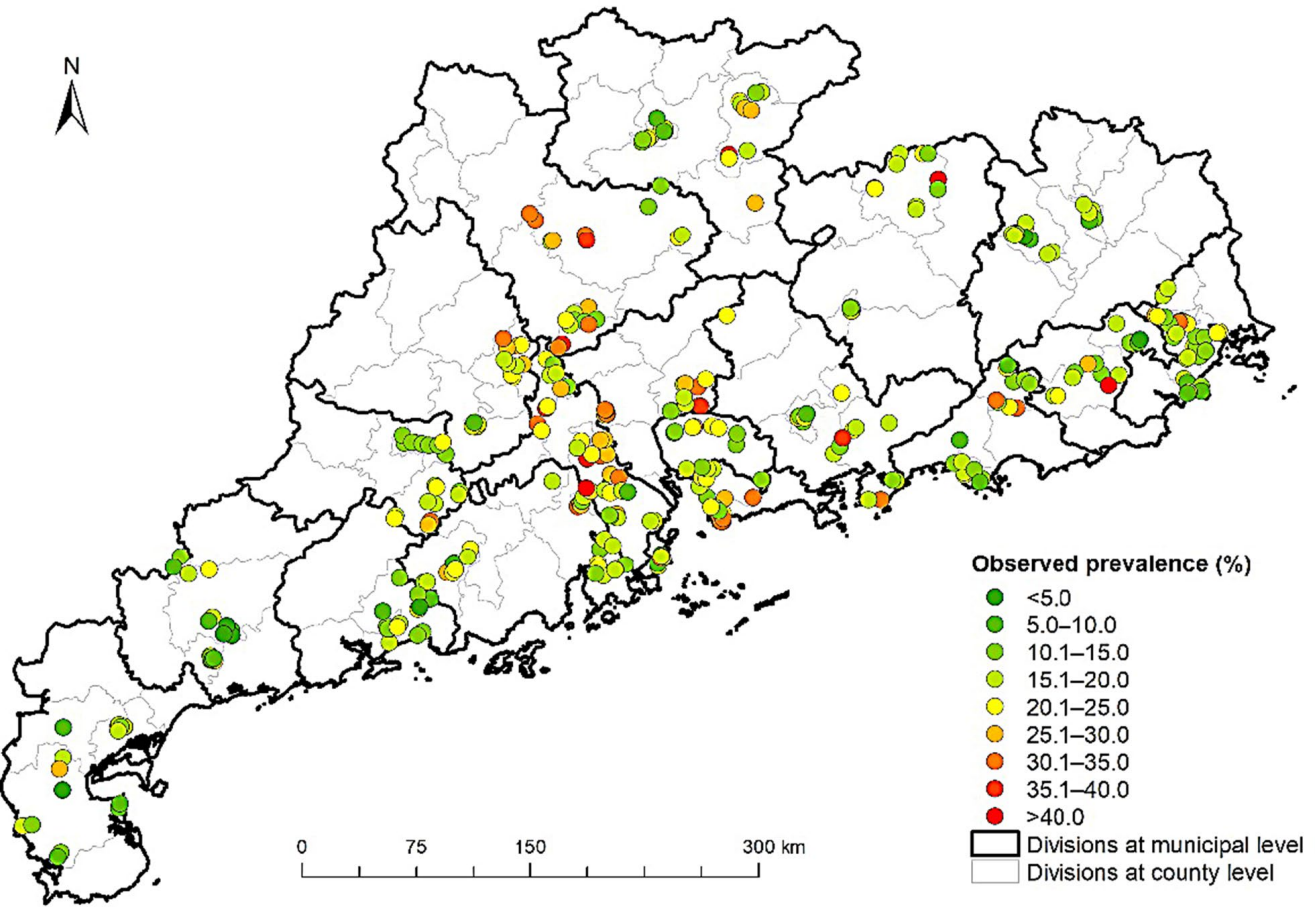
The observed two-week illness prevalence rates of each survey locations

The survey investigated 81,859 people in 24,129 households from 400 villages/communities in 40 counties across Guangdong province. The raw two-week illness prevalence was 18.5% (number of individuals who reported illness within the two-week period divided by the total number of investigated individuals). The observed prevalence for each investigated location is shown in Fig. 2. The results of the univariate analysis of individual-level variables are summarized in Table 1. We found only two variables with a correlation coefficient larger than 0.8. These were the proportion of households with unimproved drinking water and the proportion of households without sanitary toilets. These two variables came from the same data sources with similar data quality. Nevertheless, the latter variable may reflect more of the socio-economic status of households than the former one, as building sanitary toilets are usually decided by household itself, and improvement of drinking water are usually influenced by projects of local governments. Therefore, we kept the proportion of households without sanitary toilets for the subsequent analysis. 14 covariates were included in the final geostatistical model after variable selection (Table 2). People with education above the primary level had a lower illness risk than those with no education. Married people had lower risk than the unmarried ones, and people who were widowed had the highest risk. Furthermore, a higher risk was found among people who were unemployed or retired than those who were employed. The living space per capita and elevation was correlated positively with the two-week illness risk, whereas the NDVI was correlated negatively. People with higher salary per capita and locations with higher proportion of households without sanitary toilets showed high risk. Model validation revealed that our final model could correctly estimate 94.28% of the locations within the 95% BCI, and the AUC was 0.775, indicating a reasonable predictive performance. However, the positive ME of 0.61% suggested that a slightly underestimated prevalence might exist. Sensitivity analysis shows that different priors for hyperparameters had little effect on the final outcomes (Additional file 1: Table S2, Figure S5–6).

**Table 1:** Univariate analysis of individual-level covariates

Factors	Reported two-week illness (*n* = 15,178)	No reported illness (*n* = 66,681)	*p*-value
Age (years)			
< 15	1353 (8.9%)	11,574 (17.4%)	< 0.001^#,*^
15–29	898 (5.9%)	16,148 (24.2%)	
30–44	1738 (11.5%)	15,492 (23.2%)	
45–59	4262 (28.1%)	14,453 (21.7%)	
≥ 60	6927 (45.6%)	9014 (13.5%)	
Gender			
Male	7160 (47.2%)	34,427 (51.6%)	< 0.001^*^
Female	8018 (52.8%)	32,254 (48.4%)	
Ethnicity			
Han	15,092 (99.4%)	66,265 (99.4%)	0.427
Others	86 (0.6%)	416 (0.6%)	
Education level			
No education	1914 (12.6%)	2811 (4.2%)	< 0.001^#,*^
Primary	5120 (33.7%)	11,615 (17.4%)	
Junior	4000 (26.4%)	20,811 (31.2%)	
Senior	1992 (13.1%)	13,158 (19.7%)	
Bachelor or above	2152 (14.2%)	18,286 (27.4%)	
Marital status			
Unmarried	926 (6.1%)	13,118 (19.7%)	< 0.001^*^
Married	10,758 (70.9%)	39,010 (58.5%)	
Widowed	2006 (13.2%)	2093 (3.1%)	
Divorced	101 (0.7%)	440 (0.7%)	
Other	1387 (9.1%)	12,020 (18.0%)	
Employment status			
Employed	5915 (39.0%)	36,876 (55.3%)	< 0.001^*^
Unemployed	3052 (20.1%)	3749 (5.6%)	
Student	230 (1.5%)	4904 (7.4%)	
Retired	5981 (39.4%)	21,152 (31.7%)	
Occupation^$^			
Type 1	517 (3.4%)	1894 (2.8%)	< 0.001^*^
Type 2	896 (5.9%)	4369 (6.6%)	
Type 3	625 (4.1%)	2217 (3.3%)	
Type 4	1452 (9.6%)	8555 (12.8%)	
Type 5	2633 (17.3%)	10,470 (15.7%)	
Type 6	734 (4.8%)	2693 (4.0%)	
Type 7	8321 (54.8%)	36,483 (54.7%)	
Type of household registration			
Rural	9446 (62.2%)	43,909 (65.8%)	< 0.001^*^
Urban	5732 (37.8%)	22,772 (34.2%)	
Location of household registration			
In this county	14,199 (93.5%)	62,650 (94.0%)	0.048^*^
Out of this county but in this province	397 (2.6%)	1613 (2.4%)	
Out of this province	542 (3.6%)	2186 (3.3%)	
Undetermined	40 (0.3%)	232 (0.3%)	

^#^Wilcoxon rank-sum test. All other analyses were conducted using the *χ*^2^ test

^*^Significant

^$^Occupation types classified in the survey: type 1, heads of organizations, enterprises or institutions; type 2, professionals or technicians; type 3, civil servants; type 4, business/service workers; type 5, agricultural, forestry, animal husbandry, fishery or water conservancy production workers; type 6, production and transportation equipment operators; and type 7, soldiers or others

**Table 2:** Posterior summaries of the model parameters

Parameters	Estimated median (95% BCI)
Gender (male) ^#^	
Female	0.0380 (− 0.0043, 0.0803)
Education level (no education)	
Primary	0.0035 (− 0.0772, 0.0844)
Junior	− 0.0968 (− 0.1871, − 0.0063)^*^
Senior	− 0.1544 (− 0.2582, − 0.0506)^*^
Bachelor or above	− 0.1508 (− 0.2860, − 0.0160)^*^
Marital status (unmarried)	
Married	− 0.1253 (− 0.2346, − 0.0152)^*^
Widowed	0.1518 (0.0177, 0.2864)^*^
Divorced	− 0.0993 (− 0.3620, 0.1551)
Other	− 0.6890 (− 0.9716, − 0.4076)^*^
Employment status (employed)	
Unemployed	0.5886 (0.5009, 0.6763)^*^
Student	0.0153 (− 0.1935, 0.2224)
Retired	0.5290 (0.4504, 0.6078)^*^
Occupation (type 1)^$^	
Type 2	0.0228 (− 0.1183, 0.1644)
Type 3	0.1444 (− 0.0086, 0.2976)
Type 4	0.0410 (− 0.0934, 0.1763)
Type 5	0.0911 (− 0.0457, 0.2290)
Type 6	0.0648 (− 0.0888, 0.2187)
Type 7	− 0.0328 (− 0.1627, 0.0982)
Type of household registration (rural)	
Urban	0.0453 (− 0.0356, 0.1259)
Location of household registration (in this county)	
Out of this county but in this province	0.0111 (− 0.1318, 0.1519)
Out of this province	− 0.0117 (− 0.1530, 0.1283)
Undetermined	− 0.1043 (− 0.4821, 0.2466)
Living space per capita	0.2439 (0.0870, 0.4007)^*^
No-housing ratio (< 0.9%)	
0.9–2.4	0.2979 (− 0.0153, 0.6110)
2.4–4.6	0.0639 (− 0.3150, 0.4426)
≥ 4.6	− 0.0220 (− 0.3756, 0.3312)
Proportion of households without sanitary toilets (< 0.3%)	
0.3–0.7	0.0228 (− 0.2849, 0.3302)
0.7–1.6	0.2597 (− 0.0484, 0.5676)
≥ 1.6	0.4634 (0.0331, 0.8935)^*^
Travel time to cities	− 0.0767 (− 0.1687, 0.0151)
Salary per capita (< ¥ 4.0 × 10^4^/year)	
4.0–4.2	0.0511 (− 0.3374, 0.4393)
4.2–4.7	0.1760 (− 0.2763, 0.6281)
≥ 4.7	0.6969 (0.3395, 1.0540)^*^
NDVI^&^	− 0.1074 (− 0.2088, − 0.0061) ^*^
Elevation	0.1330 (0.0242, 0.2416)*
Range (km)	219.4650 (172.1405, 280.8523)
Spatial variance	1.5884 (1.0445, 2.4707)
Age correlation coefficient $$\rho$$	0.8409 (0.7579, 0.8999)
Non-spatial variance $${\delta }^{2}$$	0.1983 (0.1623, 0.2422)

^#^Baseline reference are reported in brackets

^*^Significant, determined by no inclusion of zero in the 95%BCIs

^$^Occupation types classified in the survey: type 1, heads of organizations, enterprises or institutions; type 2, professionals or technicians; type 3, civil servants; type 4, business/service workers; type 5, agricultural, forestry, animal husbandry, fishery or water conservancy production workers; type 6, production and transportation equipment operators; and type 7, soldiers or others. ^&^Normalized difference vegetation index

**Fig. 3:**
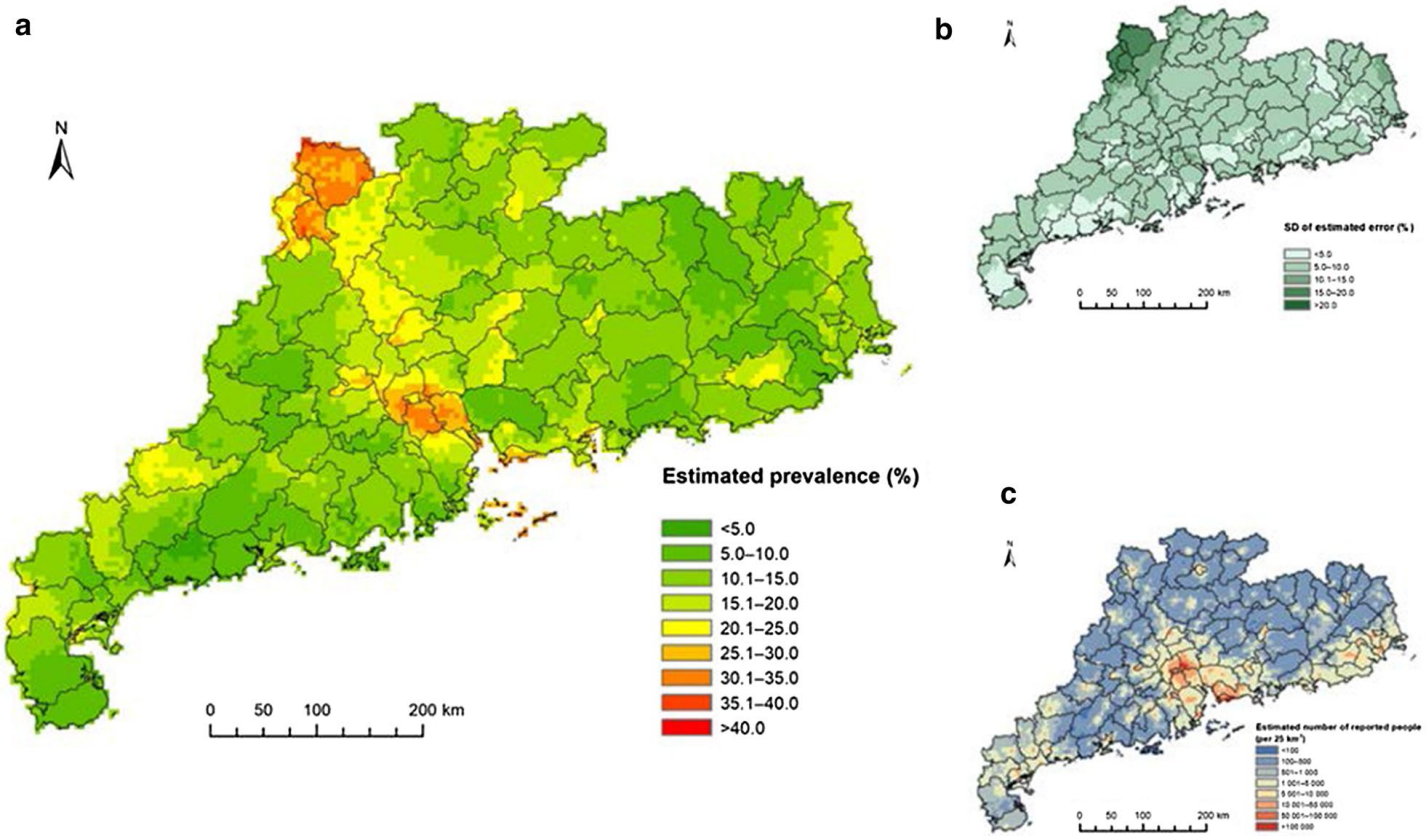
The geographical distribution of two-week illness risk in Guangdong, 2013. The maps depict results based on the median and standard deviation of the posterior predictive distribution. Estimates of **a** the two-week illness prevalence, **b** the prediction uncertainty and **c** the number of people reporting a two-week illness

The map of two-week illness prevalence (Fig. 3a) across all age groups showed notable spatial differences. The areas with the highest risk, denoted by an estimated median prevalence > 25.0%, were concentrated in the central (i.e., Pearl River Delta) and northern regions of the province, whereas many areas in southwestern and eastern regions showed the rates ranges from 10.0 to 20.0%. The highest estimated numbers of people reporting two-week illness were distributed in the Pearl River Delta and eastern coastal areas (> 10,000 people/25 km^2^) and the numbers in other areas were relatively low (mostly < 5000 people/25 km^2^) (Fig. 3c). In most areas, the standard deviation of the estimated error (Fig. 3b) ranged between 5.0 and 10.0%. Maps of random effect posteriors are shown in Additional file 1: Figure S3–4.

**Fig. 4:**
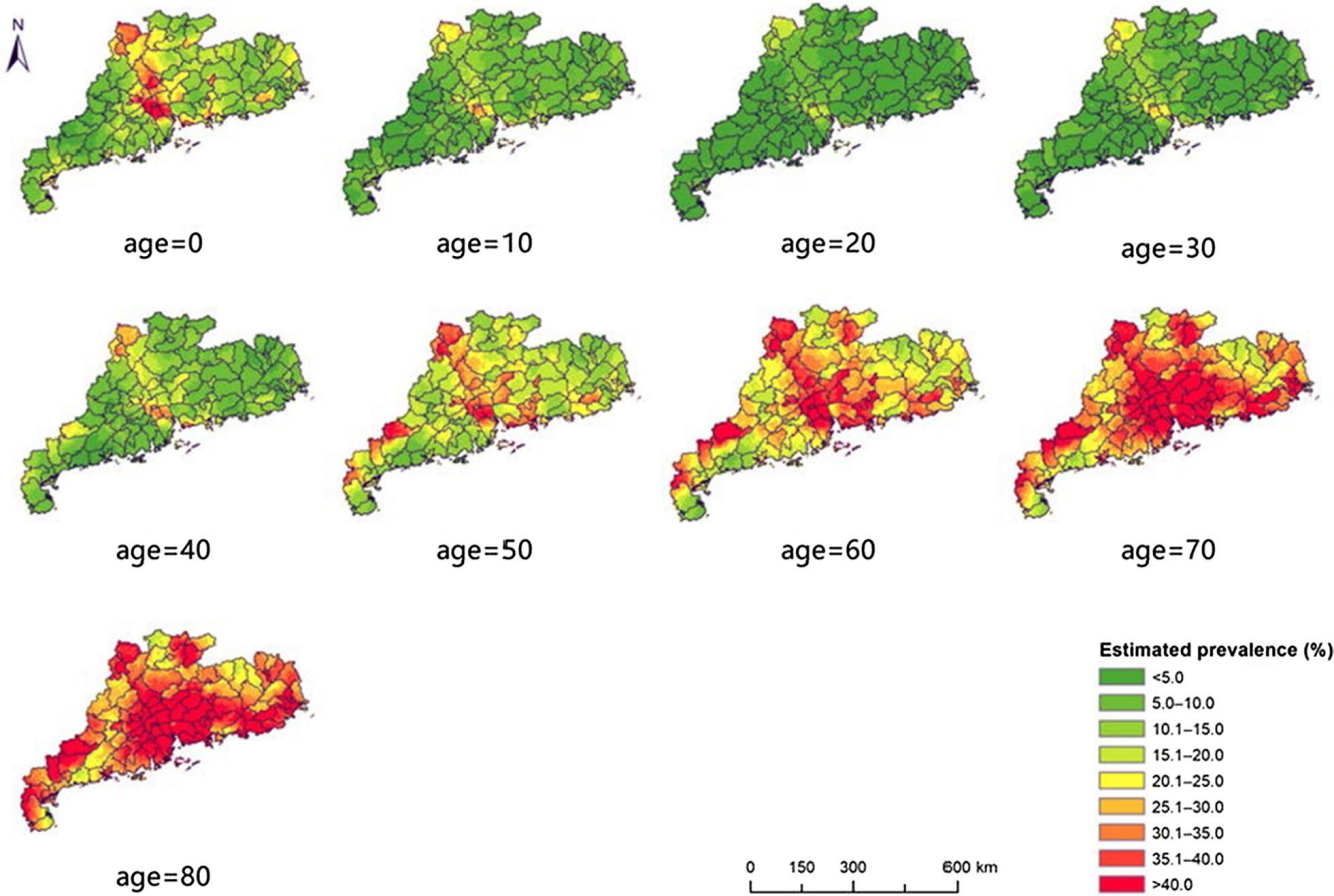
The age-specific distribution of two-week illness risk in Guangdong province, 2013. The maps depict the estimated values based on the median of the posterior predictive distribution

**Fig. 5:**
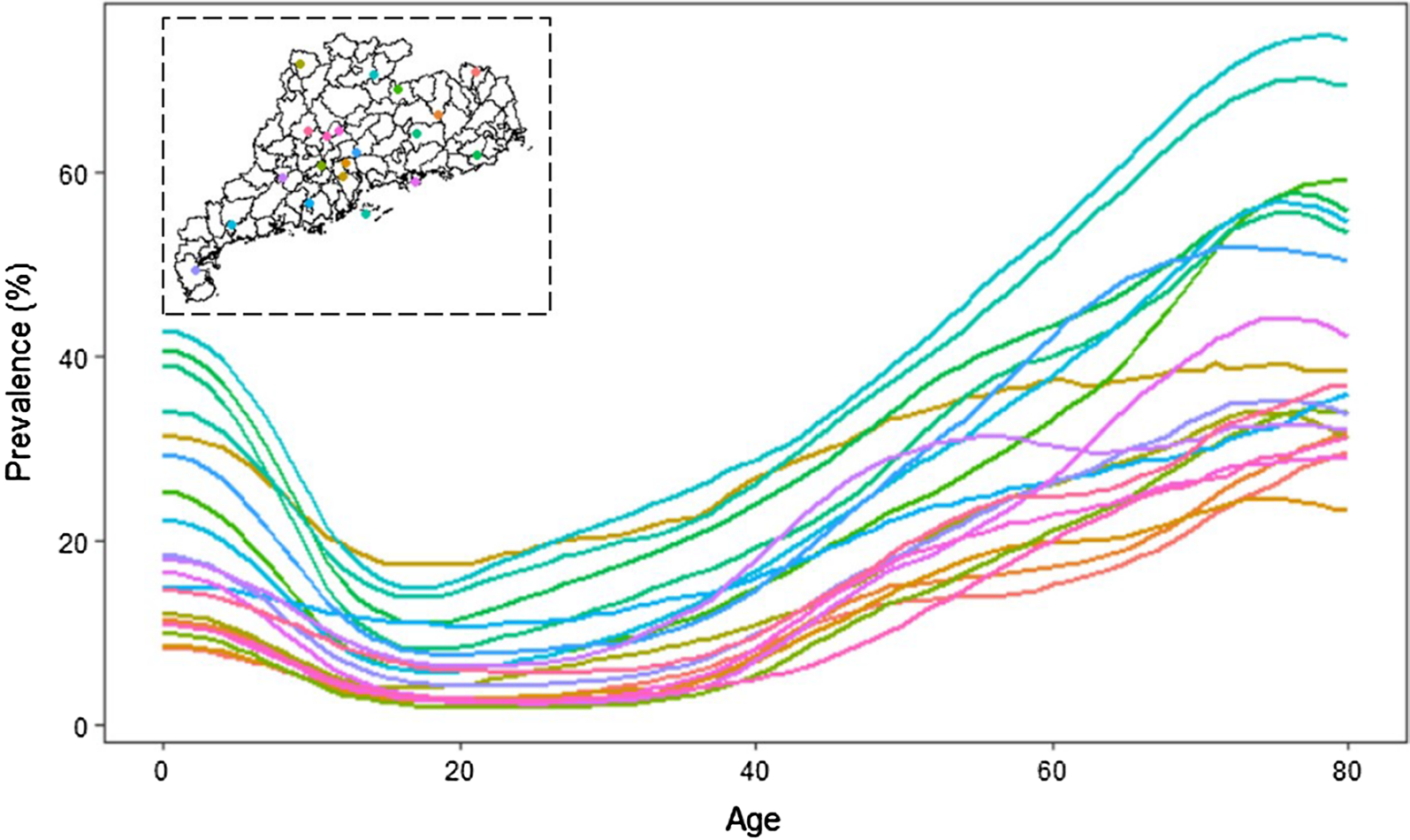
Age-two-week illness prevalence relationships (based on median of posterior predictive distribution) of 20 randomly selected locations

**Fig. 6:**
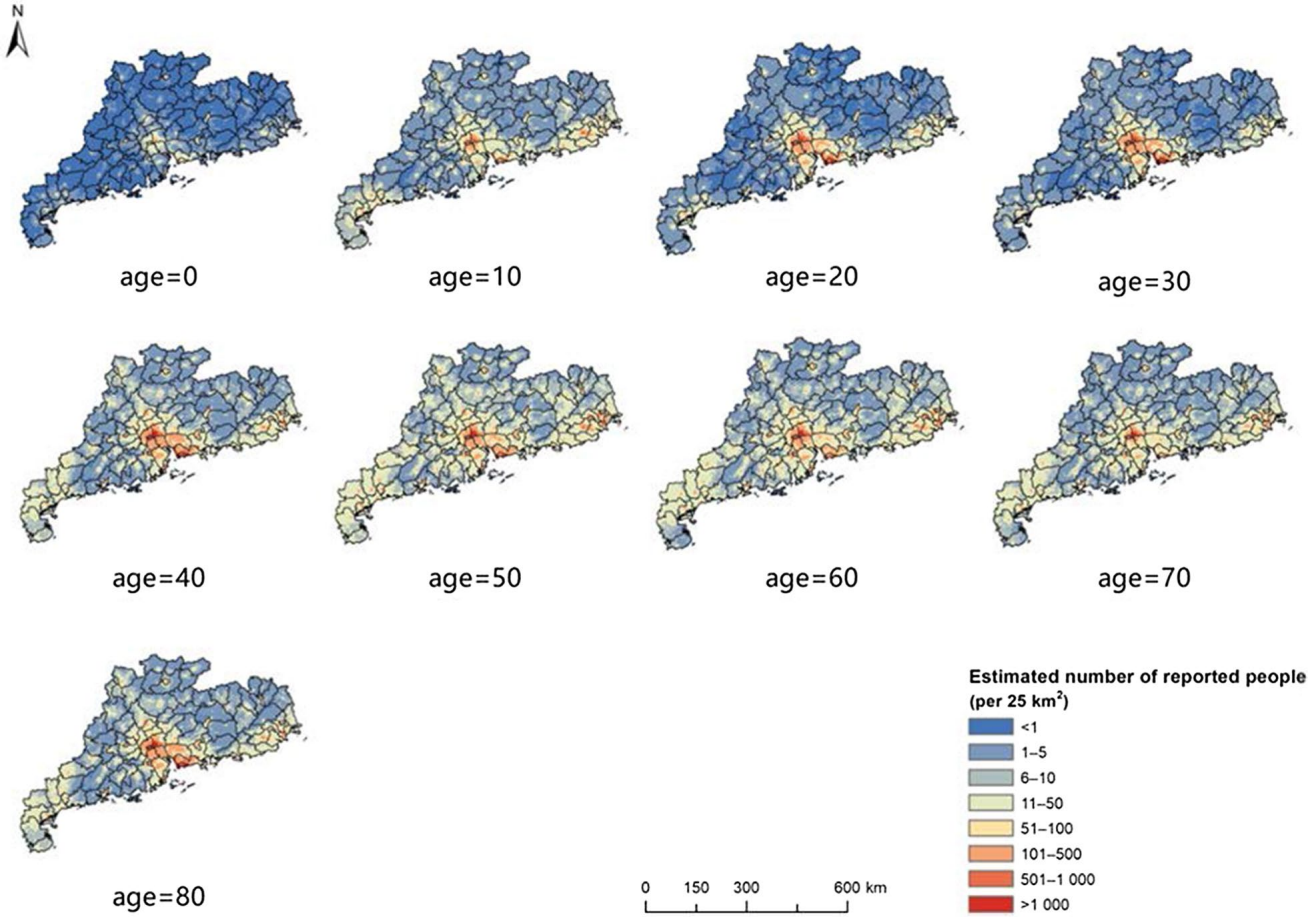
Maps of the estimated numbers of people reporting illness within two-week period for different ages in Guangdong province, 2013. The maps depict the estimated values based on the median of the posterior predictive distribution

The age-specific maps of the two-week illness prevalence (Fig. 4) had patterns similar to that of the map across all age groups with respect to spatial differences. High risk is shown in the central and northern regions, and lower risk in the southwestern and eastern regions. However, the prevalence rates differed significantly between age groups, and the age-prevalence relationships also differed across locations (Fig. 5). In most areas, the prevalence rates decrease from age 0 to age 20, and then increase gradually. The age-specific estimated numbers of people reporting illness also show spatial differences (Fig. 6), with the largest estimates in the Pearl River Delta and eastern coastal regions (> 100 people/25 km^2^ in many areas).

The estimated county-level two-week illness prevalence rates ranged from 6.4 to 32.1%. Counties with prevalence rates > 30.0% included Shunde in the central region of the province, and Liannan and Lianzhou in the northern region (Additional file 1: Table S3). Counties with larger estimated numbers of people reporting an illness within the two-week period (> 1 million) included downtown Guangzhou and uptown Shenzhen in the central region. Municipalities with higher illness prevalence rates (> 20.0%) included Foshan, Guangzhou, Qingyuan and Shenzhen, and those with larger numbers of people reporting an illness within the two-week period (> 1 million) included Foshan, Guangzhou and Shenzhen (Additional file 1: Table S4). Overall, the estimated age- and population-adjusted two-week illness prevalence in Guangdong province in 2013 was 16.5% (95% BCI: 14.5–18.6), and the total number of people reporting an illness within the two-week period was 17.5 million (95% BCI: 15.5–19.8 million).

**Fig. 7:**
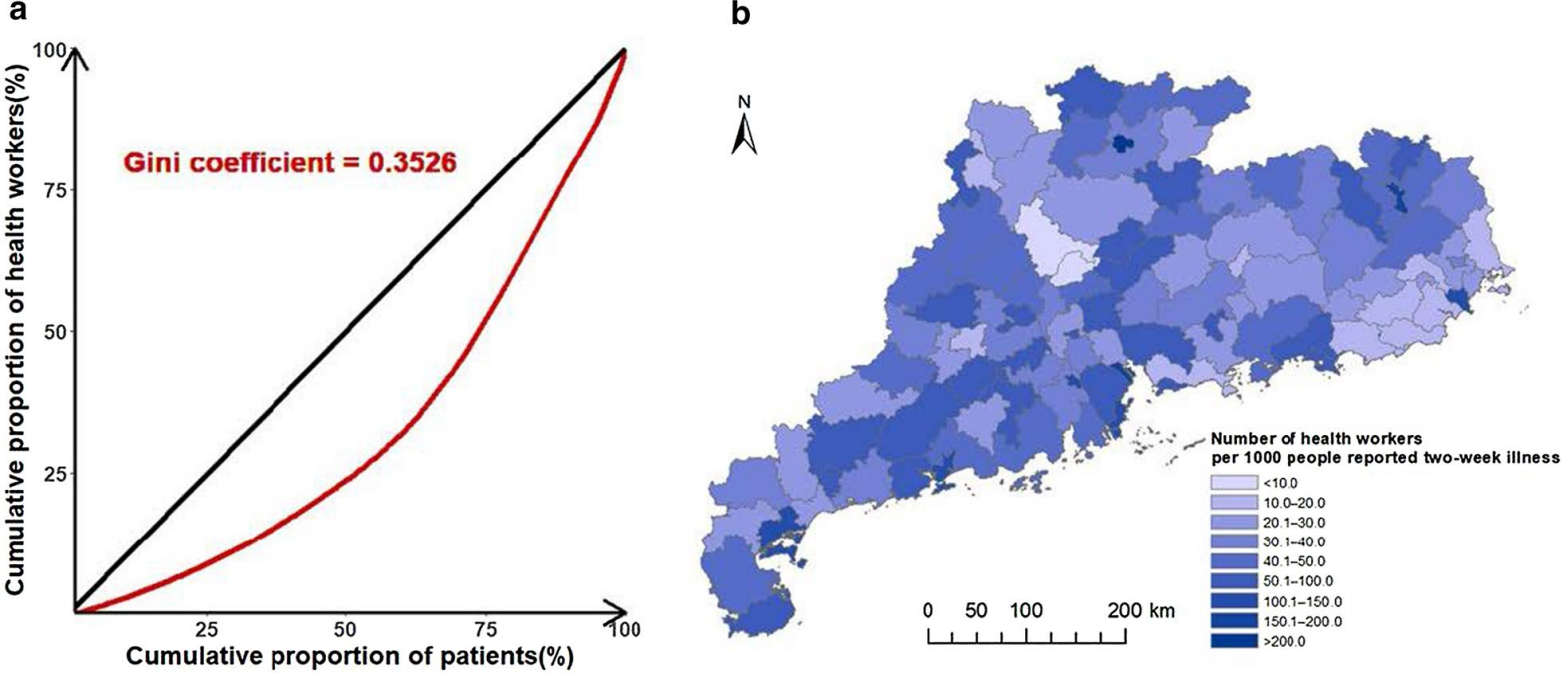
The assessment of equality in health resource distribution in Guangdong province, 2013. **a** The Lorenz curve with Gini coefficient, **b** the supply-need ratio map at county level

The Lorenz curve (Fig. 7a) and the Gini coefficient (resulted in 0.3526) showed a moderate level of inequality in health resource distribution. The supply-need ratio map called for more health resources in some counties of the northern part (Fig. 7b).

The mapping results of the major types of diseases reported show a high risk of endocrine and metabolic diseases risk in the central (i.e., Pearl River Delta) and eastern areas, a higher risk of circulatory system diseases around the central areas, a higher risk of respiratory diseases in the northern areas, and a higher risk of digestive system diseases in the central and northern regions of the study region (Additional file 1: Figure S7).

## Discussion

This study provided age-specific, high-spatial-resolution estimates of the two-week illness prevalence, an important indicator of the health levels of residents and health service needs, in Guangdong province, China. The introduction of spatial-age random effect in the Bayesian geostatistical model allows to study the specific age-prevalence relationships among different areas. Risk mapping of illness at high resolution provides information on how the risk distributed within administrative divisions, thus is useful for local policy makers. And maps at a lower resolution (e.g., county- or city-level) can be further obtained from high-resolution maps weighted by age and population, thus improving the accuracy compared to raw observed rates, and ready for use by policy makers of different levels. Moreover, information of two-week illness serves as indicator for health need, thus can be further adopted to evaluate the equality of health resource distribution. The versatile and efficient methodology can be applied to other regions if appropriate survey data are available.

To overcome the “big n problem”, we used INLA instead of the MCMC approaches for Bayesian inferences, for its fast speed and good accuracy [48]. The additional advantages include the allowance for greater automation and parallel implementation [46]. Though the application of INLA is mostly restricted to class of latent Gaussian models [48], it’s ready to use widely as most common statistical models belong to this class. Furthermore, we introduced spatial-age structure random effect to assess the space-age interaction. Separated by year, number of age groups is large. To decrease the computation burden, we built the GMRFs on equally spaced age knots and approximate the latent fields of other age groups by projection of the knot fields. It is a trade-off between calculation accuracy and calculation speed. Nevertheless, the model performance is good.

Our model was able to correctly estimate 94.28% of the locations within 95% BCI, and had an AUC of 0.775, suggesting a reasonable model performance. On one hand, the spatial-age random effect introduced in the model allows to describe age- and location-specific variations across the study region. Thus, we are able to not only acquire the age-specific and the overall prevalence rates, but also estimate the age-prevalence relationships among different areas. Compared with common surveillance reports, national health surveys usually contain specific age information on participants, which provides the chance to improve the estimation by making full use of the individual age information. Our model is ready to apply on risk estimates with similar data structure. On the other hand, we made use of both various data from the national health survey and data from other open-access databases, considering both individual- and location-level potential predictors, which may improve the prediction, compared to models only with location-specific predictors or only based on data from single source.

Our final geostatistical model identified that socio-economic predictors, such as education level, marital status, employment status, proportion of households without sanitary toilets, living space per capita and salary per capita were significantly related to the two-week illness risk. Previous studies identified low education level as a risk factor for cardiovascular diseases and metabolic syndrome [49, 50], the major endemic diseases in Guangdong province [38]. Studies show that widowhood a risk factor for dementia and Alzheimer’s disease [51, 52], unemployment could increase the risk of common mental disorders [53], and retirement may increase the risk of mental disorders and chronic diseases [54, 55]. Lower proportion of households without sanitary toilets, higher living place per capita and higher salary per capita reflects partially a better socio-economic status of households, people with which may be more sensitive to illness and likely to report the status [56, 57]. Studies also found income associated with metabolic syndrome related disorders such as obesity and type 2 diabetes [58, 59]. Taken together, these previous findings might support the corresponding associations of socio-economic factors identified in this study with the two-week illness risk. We additionally found environmental predictors, that is elevation and NDVI, with significant relationships with the two-week illness prevalence. These factors may interact health status in more complex ways, directly or indirectly [60, 61]. Our study did not identify gender, occupation, type of household registration or location of household registration as significant risk factors in the final model, and this might be partially due to the existing correlations between the covariates (Additional file 1: Table S5).

The maps of the estimated age-specific and overall two-week illness prevalence distributions showed a greater concentration of illness prevalence in the central (the Pearl River Delta) and northern regions of the province, suggesting relatively greater need of health service in these areas. The province government should fully consider the various of health need across the study region, together with the current health supply, for a more reasonable allocation of new health resources. Furthermore, as the risk maps at high spatial resolutions show the difference of health need within administrative divisions, local governments should fully consider such difference for future planning of health supply distribution within the divisions. Reasons for geographical differences in two-week illness prevalence might result from large diversity of socio-economy, behaviors and climate across the study region. As higher risk of certain chronic diseases (e.g., metabolic syndrome) was found associated with higher income [58, 59], the higher prevalence of these diseases in the Pearl River Delta, the most developed region of the province, may contribute greatly to the high risk of the two-week illness in the region. In the northern region, the cold and humid climate and poor economic conditions may encourage people to burn wet firewood for cooking, which could increase the risk of smog-related diseases [62]. Risk maps of the typical types of diseases (Additional file 1: Figure S7) were consistent with the findings. Nevertheless, more detailed investigations are needed, particularly in vulnerable areas, to further clarify the reasons of geographical differences and to guide the specific disease control management.

A study showed that in 2013, the Gini coefficient of health worker number versus the population in Guangdong province was 0.1800, drawing the conclusion of absolute equality in health resource distribution in Guangdong [63]. In contrast, our study worked out a Gini coefficient of 0.3526, concluding the moderate level of inequality in health resource distribution. Since both studies use the same indicator (i.e., number of health workers) with the same data source for the supply [42], but different indicators (i.e., population and the estimated number of people reporting two-week illness in the previous and the current studies, respectively) for the need, the discrepancy conclusions reveal the decisive effect of the selection of need indicators. The moderate level of inequality resulted in our study calls for further optimization of health resource allocation in Guangdong, and the map of supply-and-need ratio may provide useful supporting information.

The key data used in this study were obtained through the fifth National Health Services Survey in 2013, which covered 31 provinces, 156 counties, 93,613 families and 273,688 residents in China [64]. Apart from the two-week illness status, the survey collected information on a wide range of indicators, such as prevalence of different types of diseases, two-week hospital visitation rate, annual hospitalization rate and travel time to the nearest medical institution [64]. Thus, it provides a wealth of information about the needs, demands and utilization of health services nationwide, which enables follow-up studies focusing on mapping of other indicators of interest. Running separate models of acute and chronic diseases would improve the analysis considering confounders like age and help inform needs for different illness types. However, based on the questionnaire, it is difficult to make a complete distinction between those who had acute and chronic diseases. As a complement, we run separate models of the major types of diseases reported (i.e., endocrine and metabolic diseases, circulatory system diseases, respiratory diseases, and digestive system diseases), to show the potential of the methodology to study different types of illness. The risk mapping of different types of diseases informs the disease-specific health needs (Additional file 1: Figure S7).

Beyond China, many other countries have conducted health surveys that consider health service needs, demands and utilization [14, 17, 65, 66]. These surveys usually include data on the illness prevalence in a limited number of sampled locations. Accordingly, analogous studies could be conducted based on the Bayesian geostatistical modeling framework, and these analyses would provide high-resolution, age-specific estimates of the illness prevalence throughout any region of interest. We provided the corresponding R codes as Additional file 2.

Our study had several limitations. First, the self-reported illness status in the two weeks prior to the survey might have been subject to both reporting bias and recall bias. Both types of bias are inevitable because of the pre-specified survey design and outcome indicators. However, a previous study noted that self-reported data can be reasonably reliable [67]. Second, we intended to consider as many potential influencing factors as possible. To meet this goal, however, we sacrificed some of the spatial resolution. For example, some of the covariates were collected merely at the county level, and their inclusion might reduce the credibility of the estimations. Nevertheless, the model validation showed a reasonable predictive performance. Third, when this article was written, the sixth National Health Services Survey had been completed, but we were unable to obtain data at the village/community level. We will update our study accordingly when these data are available.

## Conclusions

In conclusion, we developed a Bayesian geostatistical modeling framework with spatial-age structured effect to analyze health services survey data. High-resolution and age-specific maps of the two-week illness prevalence rate and the estimated number of people reporting illness within a two-week period are provided in Guangdong province, China. The versatile and efficient methodology can be applied in any regions where the appropriate survey data are available, and results will help to support plans for health resource allocation.

## Supplementary Information

**Additional file 1.** Supplementary materials for the methods, results and discussion.

**Additional file 2.** The R codes of the study.

## Data Availability

The original individual-level dataset of the health services survey is not publicly available due to the confidentiality required by the fifth National Health Services Survey. All other data are available from the open-access databases.

## Abbreviations

GDP: Gross domestic product
LST: Land surface temperature
NDVI: Normalized difference vegetation index
TCC: Total carbon content
PM_2.5_: Particulate matter < 2.5 µm in diameter
NCEI: National Centers for Environmental Information
MODIS: Moderate resolution imaging spectroradiometer
PSL: Physical Sciences Laboratory
SRTM: Shuttle Radar Topographic Mission
REDCP: Resource and Environment Data Cloud Platform
SEDAC: Socioeconomic Data and Applications Center
ENVF: Environmental Central Facility
GMRF: Gaussian Markov Random Fields
INLA: Integrated nested Laplace approximations
BCI: Bayesian credible interval
ME: Mean error
MAE: Mean absolute error
AUC: Area under the curve
ROC: Receiver operating characteristic
