# Supplementary material for: High-resolution age-specific mapping of the two-week illness prevalence rate based on the National Health Services Survey and geostatistical analysis: a case study in Guangdong province, China
Source: Int J Health Geogr. 2021 May 3;20:20. doi: 10.1186/s12942-021-00273-1 (PMC8094611; doi:10.1186/s12942-021-00273-1)
Supplement: Supplementary file 1 — Additional file 1. Supplementary materials for the methods, results and discussion. [file 12942_2021_273_MOESM1_ESM.docx]

**Supplementary**

**Table S1.** Sources of demographic, environmental and socioeconomic data in Guangdong province.

| **Data type** | **Source** | **Data period** | **Spatial resolution** | **Temporal resolution** |
| --- | --- | --- | --- | --- |
| Population | WorldPop ^1^ | 2010, 2015 | 1 km | Yearly |
| Demographic composition (age and gender) | WorldPop ^#^ | 2010, 2015 | 1 km | Yearly |
| Age composition | GBD ^2,#^ | 2013 | Country-level | Yearly |
| Ethnicity | China Data Online ^3^ | 2010 | County-level | Yearly |
| Education level | China Data Online | 2010 | County-level | Yearly |
| Marital status | China Data Online | 2010 | County-level | Yearly |
| Employment status | China Data Online | 2010 | County-level | Yearly |
| Occupation | China Data Online | 2010 | County-level | Yearly |
| Type of household registration | China Data Online | 2010 | County-level | Yearly |
| Location of household registration | China Data Online | 2010 | County-level | Yearly |
| Living space per capita | China Data Online | 2010 | County-level | Yearly |
| Proportion of no housing population | China Data Online | 2010 | County-level | Yearly |
| Proportion of households with unimproved drinking water | China Data Online | 2010 | County-level | Yearly |
| Proportion of households without sanitary toilets | China Data Online | 2010 | County-level | Yearly |
| Family population | China Data Online | 2010 | County-level | Yearly |
| Travel time to cities | The Malaria Atlas Project ^4^ | 2015 | 1 km | Yearly |
| Gross domestic product | Guangdong Statistical Yearbook ^5^ | 2013 | County-level | Yearly |
| Salary | Guangdong Statistical Yearbook | 2013 | County-level | Yearly |
| Nightlight | NCEI ^6^ | 2013 | 1 km | Yearly |
| Landcover | MODIS ^7^/Terra | 2013 | 500 m | Yearly |
| LST ^8^ (in the daytime and at night) | MODIS/Terra | 2013 | 1 km | 8 days |
| NDVI ^9^ | MODIS/Terra | 2013 | 1 km | 16 days |
| Soil moisture | PSL ^10^ | 2013 | 60 km | Monthly |
| Elevation | SRTM ^11^ | 2000 | 1 km | Yearly |
| Air temperature | REDCP ^12^ | 2013 | 1.4 km | Yearly |
| Precipitation | REDCP | 2013 | 1.4 km | Yearly |
| Fire emissions indicators (TCC ^13^) | SEDAC ^14^ | 2013 | 30 km | Yearly |
| PM_2.5_ ^15^ | ENVF ^16^ | 2013 | 3.6 km | Yearly |

^1^ WorldPop, available at: https://www.worldpop.org/. ^2^ Global Burden of Disease, available at http://www.healthdata.org/gbd/2019. ^3^ China Data Online, available at: https://www.china-data-online.com/. ^4^ The Malaria Atlas Project, available at: https://map.ox.ac.uk/research-project/accessibility_to_cities/. ^5^ Guangdong Statistical Yearbook, available at: http://stats.gd.gov.cn/gdtjnj/index.html. ^6^ National Centers for Environmental Information (NCEI), available at: https://www.ngdc.noaa.gov/ngdc.html. ^7^ Moderate Resolution Imaging Spectroradiometer (MODIS), available at: https://modis.gsfc.nasa.gov/. ^8^ Land surface temperature. ^9^ Normalized difference vegetation index. ^10^ Physical Sciences Laboratory (PSL), available at: https://psl.noaa.gov/. ^11^ Shuttle Radar Topographic Mission (SRTM), available at: http://srtm.csi.cgiar.org/. ^12^ Resource and Environment Data Cloud Platform (REDCP), available at: http://www.resdc.cn/. ^13^ Total carbon content. ^14^ Socioeconomic Data and Applications Center (SEDAC), available at: https://sedac.ciesin.columbia.edu/. ^15^ Particulate matter <2.5 µm in diameter. ^16^ Environmental Central Facility (ENVF), available at: http://envf.ust.hk/. ^#^ The age composition from WorldPop was divided into groups every 5 years old but in grids, while the age composition from GBD was divided every 1 year old but only at country-level. Therefore, the final age composition for grids was combined by the data from these two sources.

**
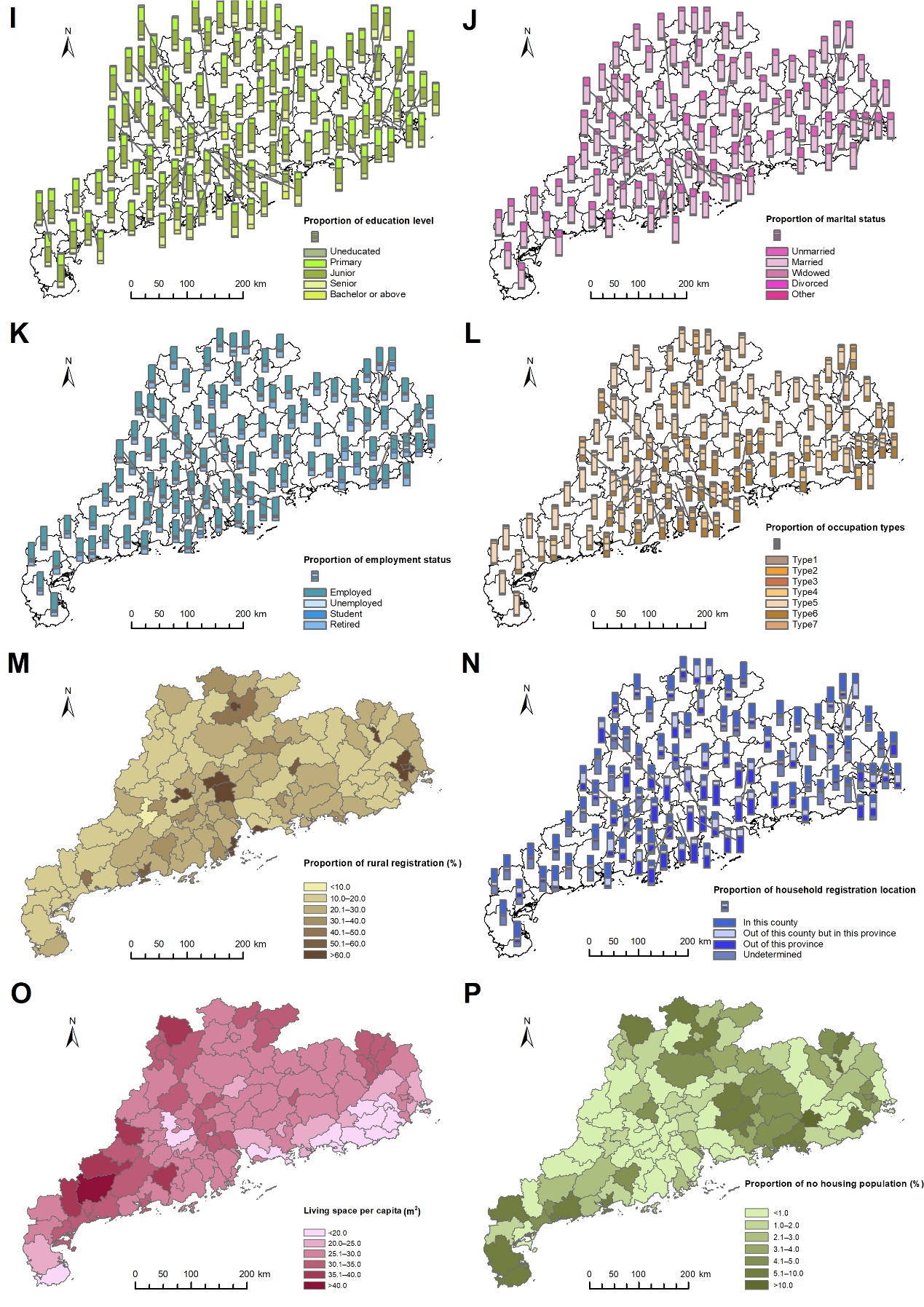

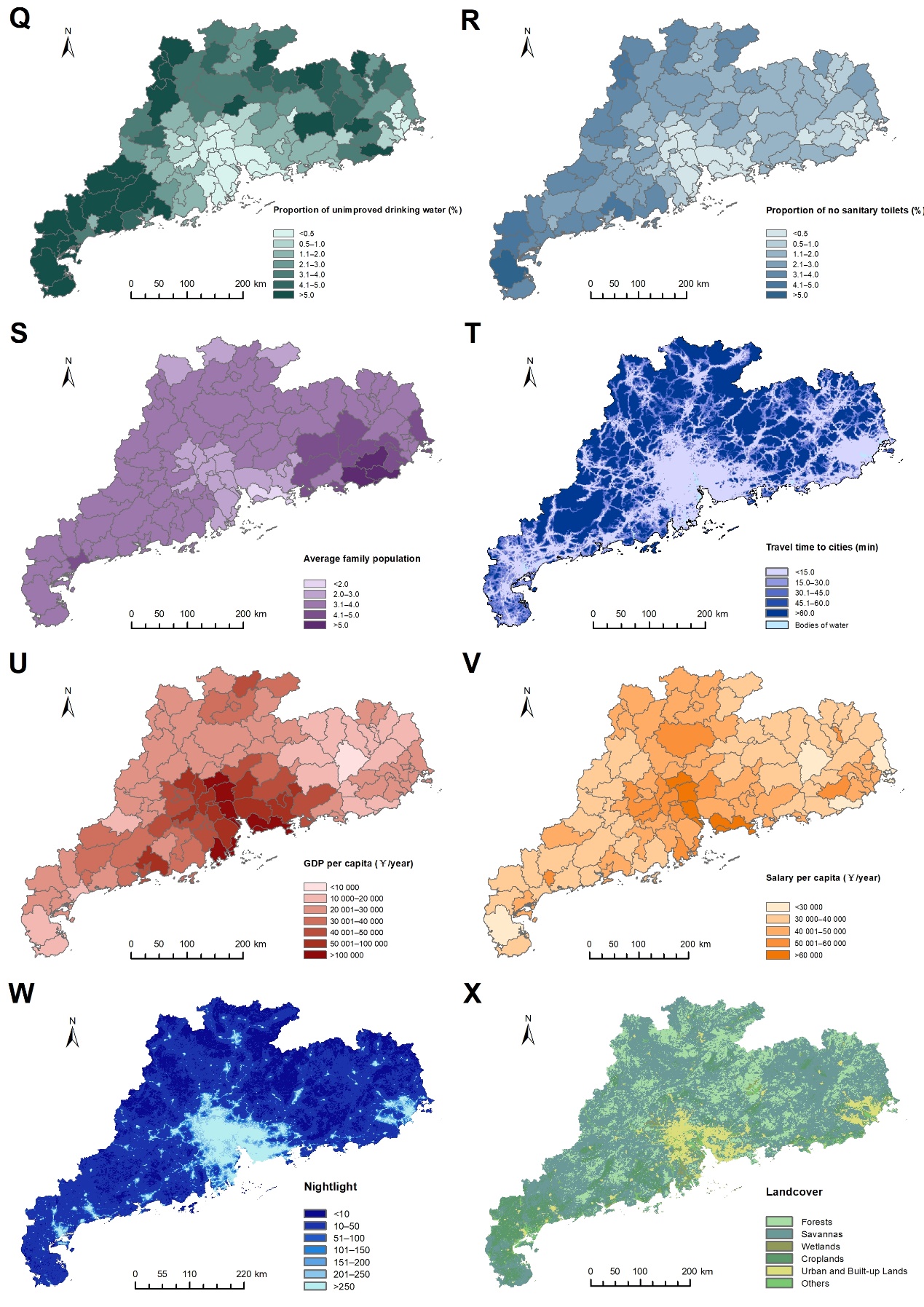
**

**Figure S1.** Images of variables used in the present study. (A) Population. (B-F) Proportion of each age group in the population. (G) Gender. (H) Ethnicity. (I) Education level. (J) Marital status. (K) Employment status. (L) Occupation (type1, heads of organs, enterprises or institutions; type 2, professionals or technicians; type 3, civil servants; type 4, business/service workers; type 5, agricultural, forestry, animal husbandry, fishery or water conservancy production workers; type 6, production and transportation equipment operators; and type 7, soldiers or others.). (M) Type of household registration. (N) Location of household registration. (O) Living space per capita. (P) Proportion of no housing population. (Q) Proportion of households with unimproved drinking water. (R) Proportion of households without sanitary toilets. (S) Family population. (T) Travel time to cities. (U) GDP per capita. (V) Salary per capita. (W) Nightlight. (X) Landcover. (Y) Land surface temperature (LST) in the daytime. (Z) LST at night. (AA) Normalized difference vegetation index (NDVI). (AB) Soil moisture. (AC) Elevation. (AD) Air temperature. (AE) Precipitation. (AF) Fire emissions indicators. (AG) PM_2.5_.

**
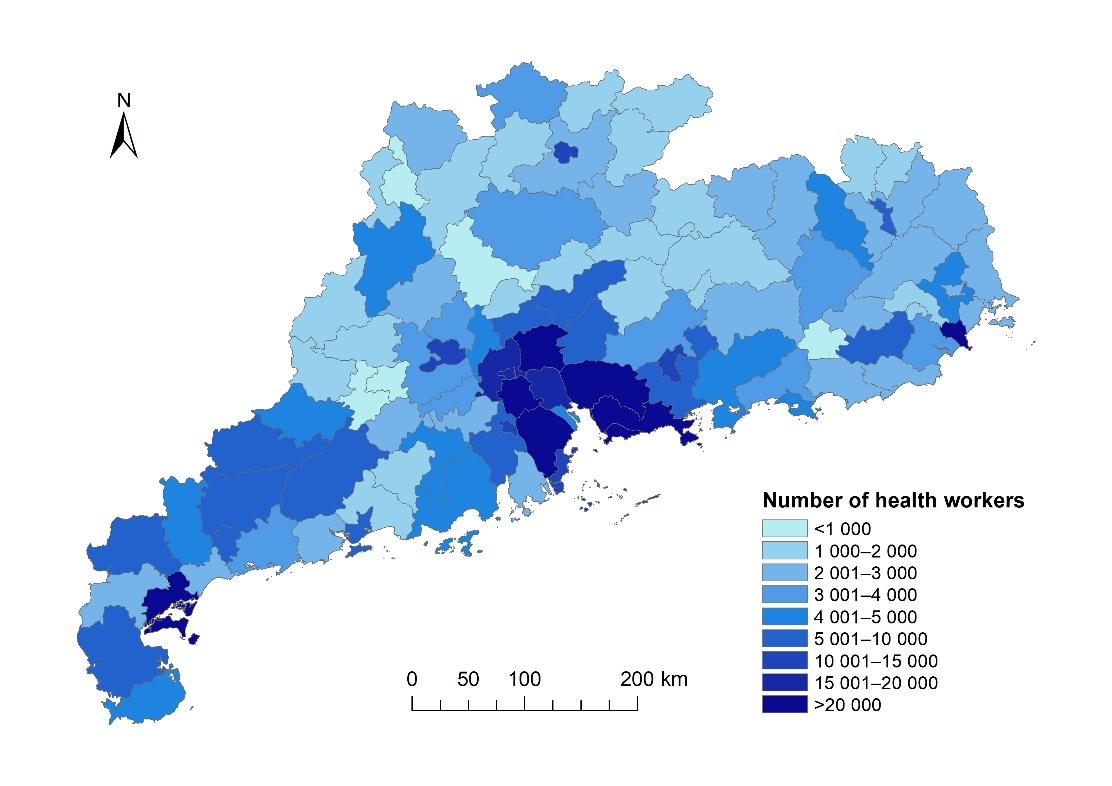
**

**Figure S2.** Number of health workers at the county level in Guangdong province, 2013.

**
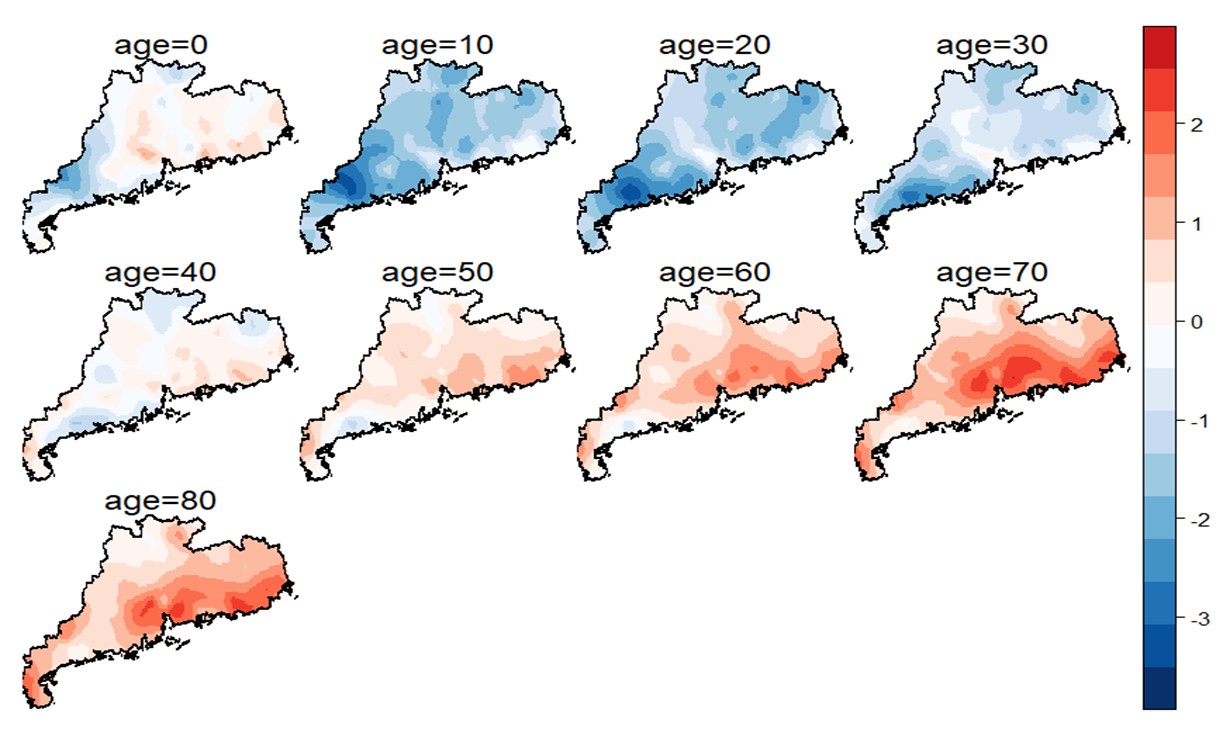
**

**Figure S3.** Posterior median of spatial-age structured random effect () of different ages.

**
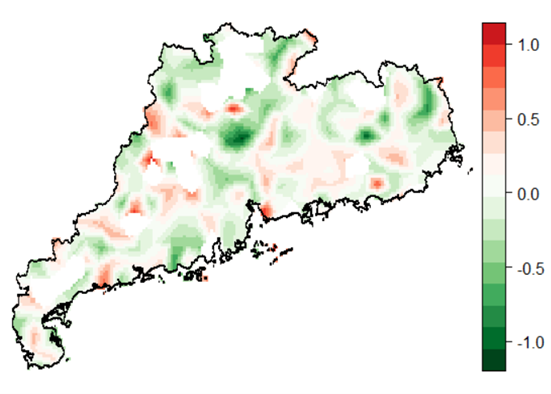
**

**Figure S4.** Posterior median of unstructured random effect ($\boldsymbol{\omega}$)

**Table S2.** Posterior medians and 95%BCIs of parameters with different priors of hyperparameters.

| **No.** | **Priors** | | | |  | | **Posteriors** | | | |
| --- | --- | --- | --- | --- | --- | --- | --- | --- | --- | --- |
|  | log(*τ*) | log(*к*) | 1/$\delta^{2}$ | $\log(\frac{1+\text{}\text{ }}{1-\text{}})$ |  | Spatial variance | | Range (km) | Non-spatial variance  ($\delta^{2}$) | Age correlation coefficient  (*ρ*) |
| 1 | *N* (0.1, 10) | *N* (1, 10) | *G* (1, 0.00005) | *N* (0, 0.15) |  | 1.5928 (1.0464, 2.4771) | | 219.6119 (172.2370, 281.1394) | 0.1981 (0.1624, 0.2422) | 0.8408 (0.7584, 0.8996) |
| 2 | *N* (1, 10) | *N* (1, 10) | *G* (1, 0.00005) | *N* (0, 0.15) |  | 1.5865 (1.0431, 2.4655) | | 219.7953 (172.6628, 281.7588) | 0.1980 (0.1624, 0.2422) | 0.8406 (0.7580, 0.8992) |
| 3 | *N* (10, 10) | *N* (1, 10) | *G* (1, 0.00005) | *N* (0, 0.15) |  | 1.5852 (1.0409, 2.4598) | | 220.3250 (173.1784, 282.7554) | 0.1979 (0.1624, 0.2424) | 0.8402 (0.7573, 0.8989) |
| 4 | *N* (1, 10) | *N* (0.1, 10) | *G* (1, 0.00005) | *N* (0, 0.15) |  | 1.5929 (1.0461, 2.4771) | | 220.6223 (173.3196, 283.1526) | 0.1979 (0.1624, 0.2424) | 0.8407 (0.7582, 0.8995) |
| 5 | *N* (1, 10) | *N* (1, 10) | *G* (1, 0.00005) | *N* (0, 0.15) |  | 1.5856 (1.0429, 2.4652) | | 219.9576 (172.6742, 281.7526) | 0.1979 (0.1624, 0.2424) | 0.8405 (0.7580, 0.8992) |
| 6 | *N* (1, 10) | *N* (10, 10) | *G* (1, 0.00005) | *N* (0, 0.15) |  | 1.5863 (1.0415, 2.4621) | | 219.5066 (172.1411, 280.9736) | 0.1979 (0.1624, 0.2424) | 0.8402 (0.7574, 0.8990) |
| 7 | *N* (1, 10) | *N* (1, 10) | *G* (0.1, 0.00005) | *N* (0, 0.15) |  | 1.5791 (1.0395, 2.4541) | | 219.7108 (172.7096, 281.7454) | 0.2000 (0.1638, 0.2444) | 0.8402 (0.7574, 0.8988) |
| 8 | *N* (1, 10) | *N* (1, 10) | *G* (1, 0.00005) | *N* (0, 0.15) |  | 1.5908 (1.0443, 2.4715) | | 219.9861 (172.6181, 281.8991) | 0.1984 (0.1623, 0.2422) | 0.8404 (0.7578, 0.8994) |
| 9 | *N* (1, 10) | *N* (1, 10) | *G* (10, 0.00005) | *N* (0, 0.15) |  | 1.6169 (1.0728, 2.5478) | | 218.5711 (170.7816, 278.3936) | 0.1809 (0.1485, 0.2214) | 0.8437 (0.7657, 0.9038) |
| 10 | *N* (1, 10) | *N* (1, 10) | *G* (1, 0.00005) | *N* (-0.5, 0.15) |  | 1.5848 (1.0408, 2.4601) | | 219.9331 (172.5144, 281.6069) | 0.1984 (0.1624, 0.2423) | 0.8400 (0.7570, 0.8988) |
| 11 | *N* (1, 10) | *N* (1, 10) | *G* (1, 0.00005) | *N* (0, 0.15) |  | 1.5833 (1.0428, 2.4638) | | 219.8153 (172.5626, 281.5118) | 0.1982 (0.1624, 0.2423) | 0.8404 (0.7581, 0.8993) |
| 12 | *N* (1, 10) | *N* (1, 10) | *G* (1, 0.00005) | *N* (0.5, 0.15) |  | 1.5900 (1.0465, 2.4763) | | 220.0440 (172.7140, 281.9333) | 0.1980 (0.1624, 0.2424) | 0.8407 (0.7589, 0.8998) |

**
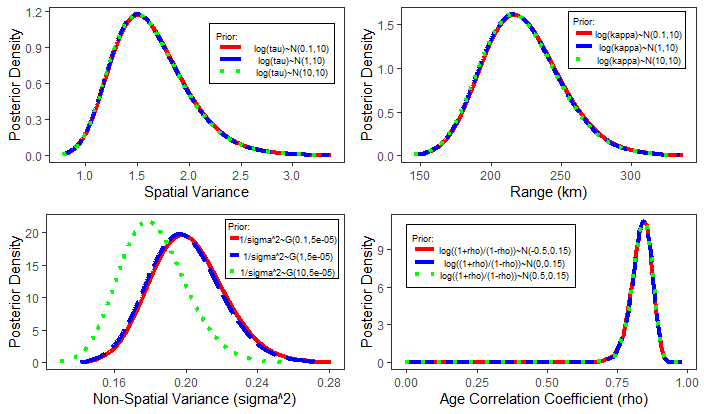
**

**Figure S5.** Posterior density distribution of parameters under different priors of hyperparameters.

**
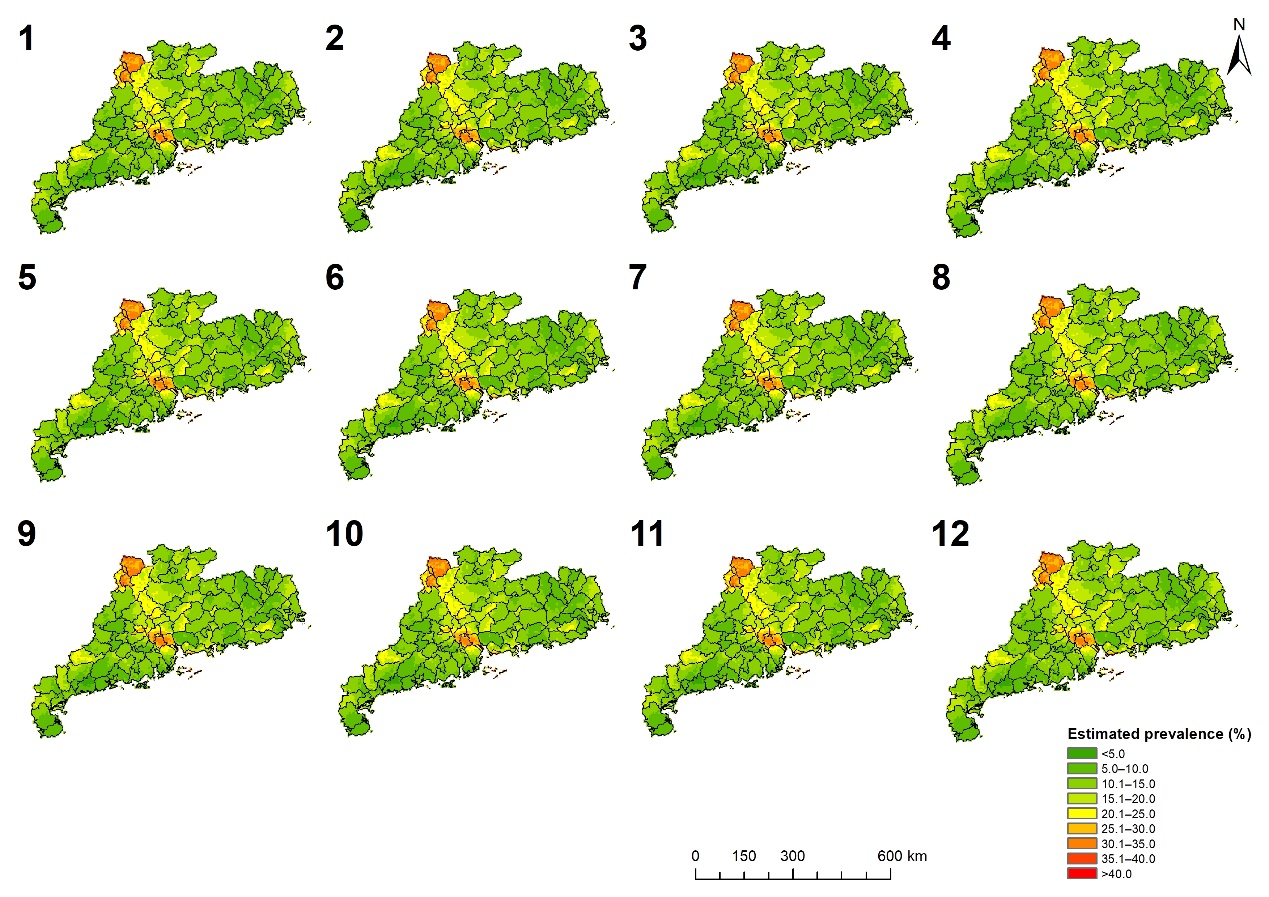
**

**Figure S6.** Mode-based estimated maps for two-week illness prevalence rate sunder different priors of hyperparameters. The maps depict the estimated values based on the median of the posterior predictive distribution. Corresponding priors are shown with the same number of indices in Table S2.

**Table S3.** Estimated age- and population-adjusted two-week illness prevalence and number of people reporting illness for each county in Guangdong province, 2013.

| **Municipality** | **County** | **Prevalence**  **(95%BCI, %)** | **Number of people**  **(95%BCI, million)** |
| --- | --- | --- | --- |
| Chaozhou | Chaonan | 14.7 (9.8, 22.6) | 0.07 (0.05, 0.11) |
| Chaozhou | downtown Chaozhou | 16.0 (10.6, 23.5) | 0.20 (0.13, 0.29) |
| Chaozhou | Raoping | 12.9 (5.1, 29.0) | 0.11 (0.04, 0.25) |
| Dongguan | Dongyuan | 9.5 (6.1, 14.1) | 0.79 (0.51, 1.16) |
| Foshan | Futian | 22.7 (14.1, 33.5) | 0.23 (0.14, 0.34) |
| Foshan | Gaoming | 15.1 (8.7, 25.0) | 0.06 (0.04, 0.10) |
| Foshan | Nanhai | 26.5 (19.0, 34.3) | 0.68 (0.49, 0.88) |
| Foshan | Sanshui | 22.0 (14.6, 31.6) | 0.13 (0.09, 0.19) |
| Foshan | Shunde | 31.0 (23.0, 39.7) ^#^ | 0.62 (0.46, 0.80) |
| Guangzhou | Conghua | 18.0 (10.5, 30.5) | 0.10 (0.06, 0.18) |
| Guangzhou | downtown Guangzhou | 24.3 (17.3, 31.9) | 1.78 (1.27, 2.34) ^$^ |
| Guangzhou | Huaxian | 19.0 (12.2, 29.7) | 0.17 (0.11, 0.27) |
| Guangzhou | Nansha | 23.9 (15.4, 33.8) | 0.02 (0.02, 0.03) |
| Guangzhou | Panyu | 29.3 (19.2, 39.9) | 0.58 (0.38, 0.79) |
| Guangzhou | Zengcheng | 20.4 (14.2, 27.6) | 0.22 (0.15, 0.29) |
| Heyuan | Dongyuan | 11.8 (6.8, 19.1) | 0.05 (0.03, 0.09) |
| Heyuan | Heping | 13.2 (8.0, 20.7) | 0.05 (0.03, 0.08) |
| Heyuan | Lianping | 13.9 (7.0, 24.4) | 0.04 (0.02, 0.08) |
| Heyuan | Lungchuan | 10.8 (6.0, 18.2) | 0.07 (0.04, 0.12) |
| Heyuan | Yuancheng | 20.7 (13.8, 29.4) | 0.09 (0.06, 0.13) |
| Heyuan | Zijin | 12.9 (7.8, 20.9) | 0.08 (0.05, 0.13) |
| Huizhou | Boluo | 12.3 (8.2, 18.8) | 0.13 (0.09, 0.20) |
| Huizhou | Huidong | 12.5 (6.9, 20.4) | 0.11 (0.06, 0.18) |
| Huizhou | Huiyang | 16.6 (11.1, 22.8) | 0.21 (0.14, 0.29) |
| Huizhou | downtown Huizhou | 12.4 (8.3, 18.1) | 0.13 (0.08, 0.18) |
| Huizhou | Longmen | 14.9 (7.6, 26.2) | 0.05 (0.02, 0.08) |
| Jiangmen | Enping | 14.5 (9.9, 20.4) | 0.07 (0.05, 0.10) |
| Jiangmen | Heshan | 11.6 (7.3, 17.4) | 0.06 (0.03, 0.08) |
| Jiangmen | downtown Jiangmen | 17.7 (11.5, 26.6) | 0.09 (0.06, 0.14) |
| Jiangmen | Kaiping | 12.0 (6.6, 20.5) | 0.08 (0.05, 0.14) |
| Jiangmen | Taishan | 12.1 (5.7, 21.4) | 0.11 (0.05, 0.20) |
| Jiangmen | Xinhui | 14.6 (10.4, 19.3) | 0.17 (0.12, 0.23) |
| Jieyang | Huilai | 13.2 (5.8, 26.2) | 0.14 (0.06, 0.27) |
| Jieyang | Jiedong | 8.7 (5.3, 14.2) | 0.10 (0.06, 0.16) |
| Jieyang | Jiexi | 9.2 (5.7, 14.0) | 0.07 (0.04, 0.11) |
| Jieyang | Puning | 19.6 (13.2, 27.6) | 0.39 (0.27, 0.56) |
| Jieyang | Rongcheng | 8.9 (5.6, 13.2) | 0.07 (0.04, 0.10) |
| Maoming | Dianbai | 6.4 (3.4, 11.5) | 0.09 (0.05, 0.16) |
| Maoming | Gaozhou | 12.1 (7.5, 18.4) | 0.15 (0.09, 0.23) |
| Maoming | Huazhou | 16.2 (7.8, 27.6) | 0.19 (0.09, 0.32) |
| Maoming | downtown Maoming | 10.0 (6.4, 15.1) | 0.09 (0.06, 0.13) |
| Maoming | Xinyi | 20.1 (11.8, 32.5) | 0.18 (0.10, 0.29) |
| Meizhou | Dapu | 16.6 (5.7, 34.3) | 0.06 (0.02, 0.12) |
| Meizhou | Fengshun | 10.3 (5.8, 16.9) | 0.05 (0.03, 0.08) |
| Meizhou | Jiaoling | 9.6 (3.7, 21.3) | 0.02 (0.01, 0.04) |
| Meizhou | Mei | 13.4 (7.9, 21.7) | 0.07 (0.04, 0.11) |
| Meizhou | downtown Meizhou | 15.0 (10.1, 20.4) | 0.06 (0.04, 0.08) |
| Meizhou | Pingyuan | 11.3 (4.5, 23.8) | 0.02 (0.01, 0.05) |
| Meizhou | Wuhua | 10.2 (5.9, 18.6) | 0.10 (0.06, 0.19) |
| Meizhou | Xingning | 8.2 (5.0, 12.9) | 0.08 (0.05, 0.12) |
| Qingyuan | Fogang | 11.1 (5.5, 19.8) | 0.03 (0.02, 0.06) |
| Qingyuan | Liannan | 30.8 (11.4, 65.6) ^#^ | 0.04 (0.01, 0.08) |
| Qingyuan | Lianshan | 25.2 (7.1, 58.4) | 0.02 (0.01, 0.06) |
| Qingyuan | Lianzhou | 32.1 (10.9, 64.9) ^#^ | 0.12 (0.04, 0.24) |
| Qingyuan | Qingcheng | 24.1 (16.2, 36.0) | 0.19 (0.13, 0.29) |
| Qingyuan | Qingxin | 21.0 (13.8, 31.3) | 0.15 (0.10, 0.22) |
| Qingyuan | Yangshan | 22.0 (10.5, 42.7) | 0.08 (0.04, 0.15) |
| Qingyuan | Yingde | 16.6 (10.9, 24.3) | 0.15 (0.10, 0.22) |
| Shantou | Chaoyang | 9.0 (5.1, 15.5) | 0.26 (0.14, 0.44) |
| Shantou | Chenghai | 13.8 (9.0, 20.6) | 0.12 (0.08, 0.18) |
| Shantou | downtown Shantou | 14.3 (9.1, 24.0) | 0.20 (0.12, 0.33) |
| Shanwei | Haifeng | 9.4 (6.4, 13.6) | 0.07 (0.05, 0.10) |
| Shanwei | Lufeng | 14.4 (7.6, 25.8) | 0.19 (0.10, 0.35) |
| Shanwei | Luhe | 13.3 (10.1, 17.0) | 0.04 (0.03, 0.05) |
| Shanwei | downtown Shanwei | 14.7 (8.9, 22.6) | 0.08 (0.05, 0.12) |
| Shaoguan | Lechang | 11.6 (4.0, 27.8) | 0.04 (0.02, 0.11) |
| Shaoguan | Nanxiong | 14.3 (5.5, 29.8) | 0.04 (0.02, 0.09) |
| Shaoguan | Qujiang | 12.8 (7.6, 20.3) | 0.07 (0.04, 0.11) |
| Shaoguan | Renhua | 16.5 (6.4, 31.9) | 0.02 (0.01, 0.04) |
| Shaoguan | Ruyuan | 15.1 (7.9, 26.7) | 0.03 (0.01, 0.05) |
| Shaoguan | downtown Shaoguan | 14.2 (8.1, 23.9) | 0.06 (0.04, 0.10) |
| Shaoguan | Shixing | 18.2 (14.0, 23.3) | 0.04 (0.03, 0.05) |
| Shaoguan | Wengyuan | 13.1 (6.6, 23.1) | 0.04 (0.02, 0.07) |
| Shaoguan | Xinfeng | 15.7 (8.0, 28.6) | 0.03 (0.02, 0.06) |
| Shenzhen | uptown Shenzhen | 17.2 (11.9, 22.7) | 1.32 (0.91, 1.74) ^$^ |
| Shenzhen | downtown Shenzhen | 27.9 (20.2, 36.1) | 0.93 (0.68, 1.21) |
| Yangjiang | Yangchun | 10.3 (5.7, 18.9) | 0.09 (0.05, 0.16) |
| Yangjiang | Yangdong | 9.9 (5.8, 15.9) | 0.04 (0.03, 0.07) |
| Yangjiang | downtown Yangjiang | 12.0 (6.5, 21.1) | 0.07 (0.04, 0.13) |
| Yangjiang | Yangxi | 6.9 (2.9, 14.4) | 0.03 (0.01, 0.06) |
| Yunfu | Luoding | 11.5 (3.8, 25.7) | 0.11 (0.03, 0.23) |
| Yunfu | Xinxing | 12.9 (8.3, 18.6) | 0.05 (0.03, 0.08) |
| Yunfu | Yun'an | 7.7 (3.8, 15.5) | 0.02 (0.01, 0.04) |
| Yunfu | Yunan | 13.6 (4.8, 28.9) | 0.05 (0.02, 0.11) |
| Yunfu | Yuncheng | 14.1 (8.6, 21.9) | 0.05 (0.03, 0.07) |
| Zhanjiang | Leizhou | 10.7 (7.5, 14.7) | 0.15 (0.11, 0.21) |
| Zhanjiang | Lianjiang | 12.0 (4.8, 24.5) | 0.17 (0.07, 0.34) |
| Zhanjiang | Suixi | 15.3 (9.6, 23.4) | 0.13 (0.08, 0.20) |
| Zhanjiang | Wuchuan | 11.1 (5.5, 21.7) | 0.10 (0.05, 0.19) |
| Zhanjiang | Xuwen | 9.4 (4.0, 18.9) | 0.06 (0.03, 0.13) |
| Zhanjiang | Zhenjiang | 13.1 (6.8, 23.8) | 0.22 (0.11, 0.40) |
| Zhaoqing | Deqing | 9.2 (4.1, 20.1) | 0.03 (0.01, 0.06) |
| Zhaoqing | Fengkai | 11.8 (3.6, 32.4) | 0.05 (0.01, 0.12) |
| Zhaoqing | Gaoyao | 15.3 (9.8, 22.7) | 0.11 (0.07, 0.17) |
| Zhaoqing | Guangning | 15.0 (7.1, 30.4) | 0.06 (0.03, 0.12) |
| Zhaoqing | Huaiji | 12.5 (4.4, 33.9) | 0.10 (0.03, 0.26) |
| Zhaoqing | Sihui | 18.6 (10.9, 30.2) | 0.09 (0.05, 0.15) |
| Zhaoqing | downtown Zhaoqing | 25.2 (16.2, 35.8) | 0.16 (0.10, 0.22) |
| Zhongshan | Zhongshan | 16.7 (12.1, 22.6) | 0.50 (0.36, 0.68) |
| Zhuhai | Doumen | 10.9 (6.6, 16.7) | 0.07 (0.04, 0.11) |
| Zhuhai | downtown Zhuhai | 14.5 (9.2, 21.3) | 0.11 (0.07, 0.17) |

^#^ Prevalence >30.0%. ^$^ Number of reported people >1 million,

**Table S4.** Estimated age- and population-adjusted two-week illness prevalence and number of people reporting illness for each municipality in Guangdong province, 2013.

| **Municipality** | **Population (million)** | **Prevalence (95%BCI, %)** | **Number of people (95%BCI, million)** |
| --- | --- | --- | --- |
| Chaozhou | 2.71 | 15.0 (10.9, 20.8) | 0.39 (0.28, 0.53) |
| Dongguan | 8.32 | 9.5 (6.1, 14.1) | 0.79 (0.51, 1.16) |
| Foshan | 7.30 | 26.3 (19.6, 33.0) ^#^ | 1.74 (1.30, 2.19) ^$^ |
| Guangzhou | 12.93 | 24.2 (18.0, 30.6) ^#^ | 2.89 (2.16, 3.67) ^$^ |
| Heyuan | 3.04 | 13.9 (9.9, 18.6) | 0.40 (0.28, 0.53) |
| Huizhou | 4.70 | 14.0 (10.9, 17.2) | 0.64 (0.50, 0.78) |
| Jiangmen | 4.50 | 13.8 (10.2, 18.1) | 0.59 (0.44, 0.78) |
| Jieyang | 5.99 | 13.6 (9.7, 18.6) | 0.77 (0.55, 1.06) |
| Maoming | 6.01 | 12.6 (9.0, 16.6) | 0.71 (0.50, 0.93) |
| Meizhou | 4.31 | 11.5 (8.3, 15.3) | 0.47 (0.34, 0.63) |
| Qingyuan | 3.79 | 22.2 (16.0, 29.9) ^#^ | 0.80 (0.58, 1.08) |
| Shantou | 5.48 | 11.4 (7.6, 16.9) | 0.58 (0.38, 0.86) |
| Shanwei | 2.99 | 13.1 (8.9, 19.4) | 0.38 (0.25, 0.56) |
| Shaoguan | 2.89 | 14.6 (10.2, 20.3) | 0.40 (0.28, 0.55) |
| Shenzhen | 10.63 | 20.4 (15.2, 25.8) ^#^ | 2.24 (1.67, 2.83) ^$^ |
| Yangjiang | 2.48 | 10.3 (6.9, 14.6) | 0.24 (0.16, 0.34) |
| Yunfu | 2.43 | 12.4 (7.2, 20.8) | 0.28 (0.16, 0.48) |
| Zhanjiang | 7.17 | 12.3 (8.2, 17.9) | 0.85 (0.56, 1.24) |
| Zhaoqing | 4.02 | 16.3 (11.4, 24.4) | 0.60 (0.42, 0.91) |
| Zhongshan | 3.17 | 16.7 (12.1, 22.6) | 0.50 (0.36, 0.68) |
| Zhuhai | 1.59 | 12.9 (9.2, 17.8) | 0.18 (0.13, 0.25) |
| Total | 106.4 | 16.5 (14.5, 18.6) | 17.5 (15.5, 19.8) |

^#^ Prevalence >20.0%. ^$^ Number of reported people >1 million,

**Table S5.** Correlation analysis of individual level covariates from the fifth National Health Services Survey in Guangdong province, 2013.

| **Covariates** |  | ***χ* ^2^** | ***p*-value**^#^ | **Cramer’s V** |
| --- | --- | --- | --- | --- |
| Age | Gender | 239.001 | <0.001 | 0.054 |
| Age | Education level | 69789.342 | <0.001 | 0.462^*^ |
| Age | Marital status | 131305.681 | <0.001 | 0.633^*^ |
| Age | Employment status | 75204.194 | <0.001 | 0.553^*^ |
| Age | Occupation | 19794.727 | <0.001 | 0.246 |
| Age | Type of household registration | 577.317 | <0.001 | 0.084 |
| Age | Location of household registration | 2166.030 | <0.001 | 0.094 |
| Gender | Education level | 2495.583 | <0.001 | 0.175 |
| Gender | Marital status | 1974.104 | <0.001 | 0.155 |
| Gender | Employment status | 680.446 | <0.001 | 0.091 |
| Gender | Occupation | 611.333 | <0.001 | 0.086 |
| Gender | Type of household registration | 0.908 | 0.341 | 0.003 |
| Gender | Location of household registration | 20.357 | <0.001 | 0.016 |
| Education level | Marital status | 63523.241 | <0.001 | 0.440^*^ |
| Education level | Employment status | 24973.839 | <0.001 | 0.319^*^ |
| Education level | Occupation | 16231.112 | <0.001 | 0.223 |
| Education level | Type of household registration | 4232.335 | <0.001 | 0.227 |
| Education level | Location of household registration | 921.403 | <0.001 | 0.061 |
| Marital status | Employment status | 61395.059 | <0.001 | 0.500^*^ |
| Marital status | Occupation | 17567.944 | <0.001 | 0.232 |
| Marital status | Type of household registration | 363.099 | <0.001 | 0.067 |
| Marital status | Location of household registration | 1285.517 | <0.001 | 0.072 |
| Employment status | Occupation | 48902.380 | <0.001 | 0.446^*^ |
| Employment status | Type of household registration | 7857.440 | <0.001 | 0.310^*^ |
| Employment status | Location of household registration | 769.335 | <0.001 | 0.056 |
| Occupation | Type of household registration | 10425.147 | <0.001 | 0.357^*^ |
| Occupation | Location of household registration | 2354.452 | <0.001 | 0.098 |
| Type of household registration | Location of household registration | 122.208 | <0.001 | 0.039 |

^#^ *p* < 0.05 denotes significant. ^*^ Correlated.

**
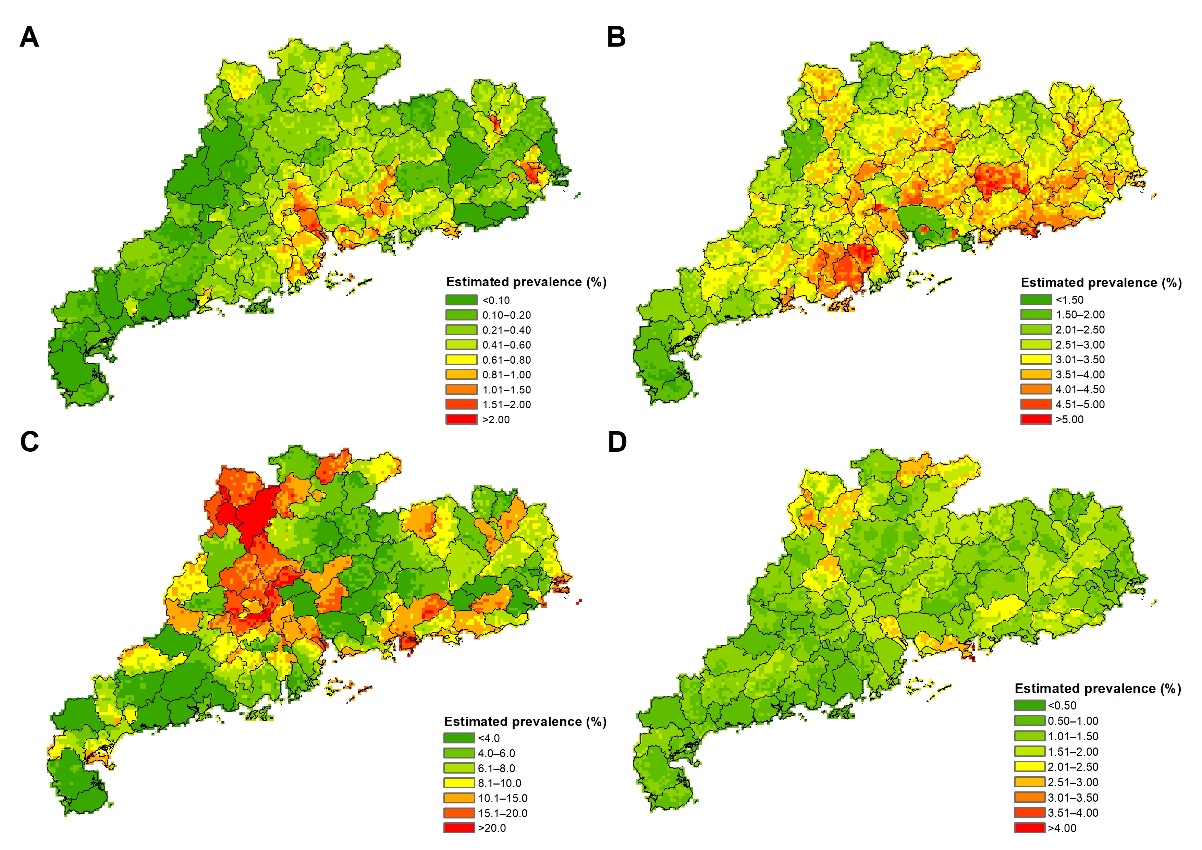
**

**Figure S7.** Estimated risk maps for different types of diseases. The maps depict the estimated values based on the median of the posterior predictive distribution. Estimates of (A) endocrine and metabolic diseases, (B) circulatory system diseases, (C) respiratory diseases and (D) digestive system diseases.
